# Exogenous Application of ENOD40 and CEP1 Peptides Boosts Symbiotic Signaling Gene Expression and Productivity in Common Bean

**DOI:** 10.3390/plants14172786

**Published:** 2025-09-05

**Authors:** Hector Cántaro-Segura, Doris Zúñiga-Dávila

**Affiliations:** 1 Facultad de Agronomía, Universidad Nacional Agraria La Molina, Lima 15024, Peru; 2 Laboratorio de Ecología Microbiana y Biotecnología, Departamento de Biología, Facultad de Ciencias, Universidad Nacional Agraria La Molina, Lima 15024, Peru

**Keywords:** plant peptides, symbiosis, common bean, signaling, nodulation, sustainable agriculture

## Abstract

Small signaling peptides play crucial roles in the regulation of legume–rhizobia symbiosis, yet their potential as exogenous biostimulants remains largely unexplored. In this study, we evaluated the effects of foliar application of the synthetic peptides ENOD40 and CEP1 on common bean (*Phaseolus vulgaris*) under both greenhouse and field conditions. Using a factorial design, we examined gene expression patterns, nodulation parameters, and yield-related traits in response to peptide treatments alone or in combination with *Rhizobium*. Results showed that ENOD40 and CEP1 significantly enhanced the transcription of key symbiotic signaling genes (*PvENOD40*, *PvSYMRK*, *PvCCaMK*, *PvCYCLOPS*, *PvVAPYRIN*) and modulated defense-related genes (*PvAOS*, *PvICS*), with the strongest effects observed at concentrations of 10^−7^ M and 10^−8^ M. In greenhouse assays, peptide-treated plants exhibited increased root and shoot biomass, nodule number, and seed yield. Field trials confirmed these responses, with CEP1 10^−7^ M + *Rhizobium* treatment achieving the highest grain yield (3322 kg ha^−1^). Our findings provide the first evidence that ENOD40 and CEP1 peptides can function as foliar-applied biostimulants to enhance nodulation efficiency and improve yield in legumes. This approach offers a promising and sustainable strategy to reduce chemical nitrogen inputs and support biological nitrogen fixation in agricultural systems.

## 1. Introduction

Leguminous crops play a vital role in sustainable agriculture [1,2] due to their ability to establish mutualistic associations with nitrogen-fixing rhizobia [3] and arbuscular mycorrhizal fungi (AMF) [4,5], which enhance nutrient acquisition—particularly nitrogen and phosphorus—improve soil fertility, and reduce reliance on synthetic fertilizers [4,6,7]. Among these, *Phaseolus vulgaris* L. (common bean) stands out as the most important grain legume for direct human consumption and a key contributor to food security [8,9], especially in low-input agricultural systems across Latin America, Africa, and Asia [10]. Its symbiotic relationship with rhizobia significantly decreases the need for external nitrogen inputs, supporting environmentally sustainable farming practices [11,12,13]. However, the efficiency of these interactions is often hampered by suboptimal nodulation, environmental stresses, and the limited regulatory capacity of the plant to sustain a successful symbiotic dialogue, ultimately restricting nitrogen fixation and productivity in nutrient-poor or stress-prone environments [3,14].

The establishment of root nodule symbiosis is orchestrated by the Common Symbiotic Signaling Pathway (Sym pathway), a highly conserved molecular cascade shared by both rhizobial and arbuscular mycorrhizal (AM) symbioses [15,16,17]. This pathway involves a core set of genes, including *SYMRK* [18,19], *CCaMK* [20], *CYCLOPS* [21,22], and *VAPYRIN* [23], that are activated upon perception of microbial signals (Nod or Myc factors), initiating downstream responses such as calcium spiking, transcriptional reprogramming, and cortical cell division [24]. In legumes, this symbiotic signaling is tightly coordinated with hormonal pathways and immune responses, highlighting a finely tuned regulatory mechanism that enables beneficial microbial colonization while preventing pathogenic invasion, suggesting a delicate balance between symbiosis and defense [7,25,26,27].

Alongside this genetic framework, plants also rely on a wide array of small regulatory peptides, which have emerged as pivotal signaling molecules in developmental and stress-response processes [28]. Plant peptide hormones, often consisting of 5 to 20 amino acids, form a functionally diverse and molecularly distinct class of signals involved in meristem maintenance, vascular differentiation, reproductive development, and the modulation of biotic and abiotic stress responses [29,30,31]. Well-known families such as CLE (CLAVATA3/ESR-related), CEP (C-terminally Encoded Peptides), PSK (Phytosulfokines), RALF (Rapid Alkalinization Factor), and IDA (Inflorescence Deficient in Abscission) illustrate the range of actions that small peptides can perform in plant growth and immunity [32]. These peptides act as mobile signals, often traveling between roots and shoots, and mediate precise physiological outcomes through receptor-like kinases, integrating local and systemic cues [31].

Among this growing class of functional peptides, ENOD40 and CEP1 have been recognized for their crucial roles in symbiotic and nutritional signaling [33,34]. ENOD40 is an early nodulin gene activated during rhizobial infection, and its peptide product is involved in the early stages of nodule organogenesis, promoting cortical cell division and influencing hormonal pathways [35,36,37]. CEP1 peptides are upregulated under nitrogen deficiency and participate in long-distance signaling to enhance nodulation through a shoot-to-root regulatory circuit [38,39]. While both peptides have been extensively studied at the transcriptomic and physiological levels, there is a remarkable gap regarding their exogenous application as biostimulants, particularly in relation to their influence on the expression of genes within the Sym pathway [40].

To our knowledge, this study represents the first report evaluating the effect of the synthetic peptides ENOD40 and CEP1 on the expression of symbiotic signaling genes in *Phaseolus vulgaris*. It is also the first investigation to assess their exogenous foliar application under both greenhouse and field conditions, integrating molecular, physiological, and agronomic assessments. This innovative approach seeks to bridge fundamental molecular biology with practical agronomic strategies, exploring whether these small peptides can enhance nodulation signaling and performance in a controlled, targeted manner.

In this context, the present study aimed to analyze the transcriptional and phenotypic effects of exogenously applied ENOD40 and CEP1 peptides, both alone and in combination with rhizobia. We evaluated their impact on the expression of core Sym pathway genes (*ENOD40*, *SYMRK*, *CCaMK*, *CYCLOPS*, and *VAPYRIN*) and plant defense-related genes (AOS and ICS), and subsequently assessed their influence on nodulation and yield components in greenhouse- and field-grown plants. The findings offer new insights into the functional deployment of signaling peptides as biotechnological tools for legume improvement, with implications for sustainable nitrogen management in agriculture [41].

## 2. Results

### 2.1. Exogenous Application of ENOD40 and CEP1 Peptides Activates Symbiotic Signaling Genes in Common Bean Roots

The application of synthetic peptides ENOD40 and CEP1 significantly modulated the expression of key genes involved in the common symbiotic signaling pathway in *Phaseolus vulgaris*.

**Figure 1:**
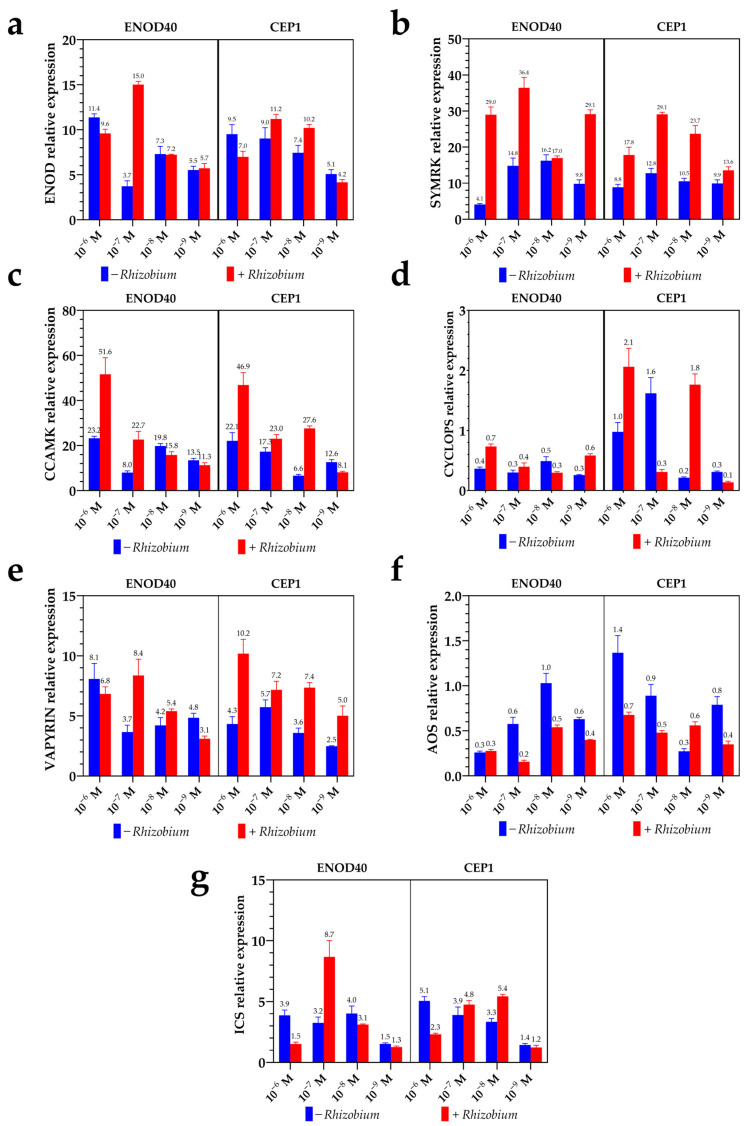
Relative expression of symbiotic genes in *Phaseolus vulgaris* roots in response to foliar application of ENOD40 and CEP1 peptides with and without *Rhizobium tropici* inoculation: (**a**) *PvENOD40*, (**b**) *PvSYMRK*, (**c**) *PvCCaMK*, (**d**) *PvCYCLOPS*, (**e**) *PvVAPYRIN,* (**f**) *PvAOS* and (**g**) *PvICS*. Bars represent mean ± standard error of three biological replicates.

These genes include *PvENOD40*, *PvSYMRK*, *PvCCaMK*, *PvCYCLOPS*, and *PvVAPYRIN*, all of which are known to play crucial roles in the early stages of root nodule formation and the establishment of rhizobial symbiosis. The gene *PvENOD40* exhibited a robust transcriptional response, with its highest expression observed in plants treated with ENOD40 at 10^−7^ M combined with *Rhizobium*, resulting in a 15-fold increase compared to the control. CEP1 at 10^−7^ M + *Rhizobium* also enhanced *PvENOD40* expression significantly (11.2-fold), although to a lesser extent than ENOD40 itself. In the absence of *Rhizobium*, both peptides still increased *PvENOD40* transcript levels, but the magnitude of response was consistently lower, highlighting the synergistic effect of peptide and rhizobial signals in activating this gene (Figure 1a).

The receptor-like kinase *PvSYMRK*, a central component of Nod factor perception and signal transduction, responded strongly to both peptides. The highest expression level (36.4-fold) was detected in the ENOD40 10^−7^ M + *Rhizobium* treatment, while CEP1 at 10^−6^ M + *Rhizobium* induced a 29-fold increase, similar to that obtained with CEP1 at 10^−9^ M + *Rhizobium* (29.1-fold) (Figure 1b). This pattern was echoed in the expression of *PvCCaMK*, a calcium/calmodulin-dependent protein kinase essential for decoding calcium oscillations during symbiosis, which peaked in ENOD40 10^−6^ M + *Rhizobium* treatment (51.6-fold), similar to the result obtained with CEP 10^−6^ M + *Rhizobium* (46.9-fold); in contrast, treatments at lower concentrations resulted in weaker effects (Figure 1c).

The transcription factors *PvCYCLOPS* and *PvVAPYRIN*, which act downstream of *CCaMK* and facilitate nodule development and infection thread progression, exhibited variable responses. Notably, *PvCYCLOPS* was consistently downregulated by ENOD40 across all tested concentrations, whereas CEP1 induced a more variable effect (Figure 1d); meanwhile, PvVAPYRIN was upregulated with the application of ENOD40 and CEP1, but with variable effects at different concentrations (Figure 1e).

### 2.2. Peptide Application Suppresses Jasmonate-Associated Defense Gene Expression Without Strongly Affecting Salicylate Pathways

In addition to modulating symbiotic gene expression, the application of the synthetic peptides ENOD40 and CEP1 also influenced the transcriptional regulation of defense-related genes. Specifically, two key genes involved in phytohormone-mediated plant immunity—*PvAOS* (*Allene Oxide Synthase*) and *PvICS* (*Isochorismate Synthase*)—were evaluated to determine whether peptide signaling impacts defense responses during the early stages of rhizobial interaction. These genes are central to the biosynthesis of jasmonic acid (JA) and salicylic acid (SA), respectively, which coordinate distinct branches of plant immunity.

**Figure 2:**
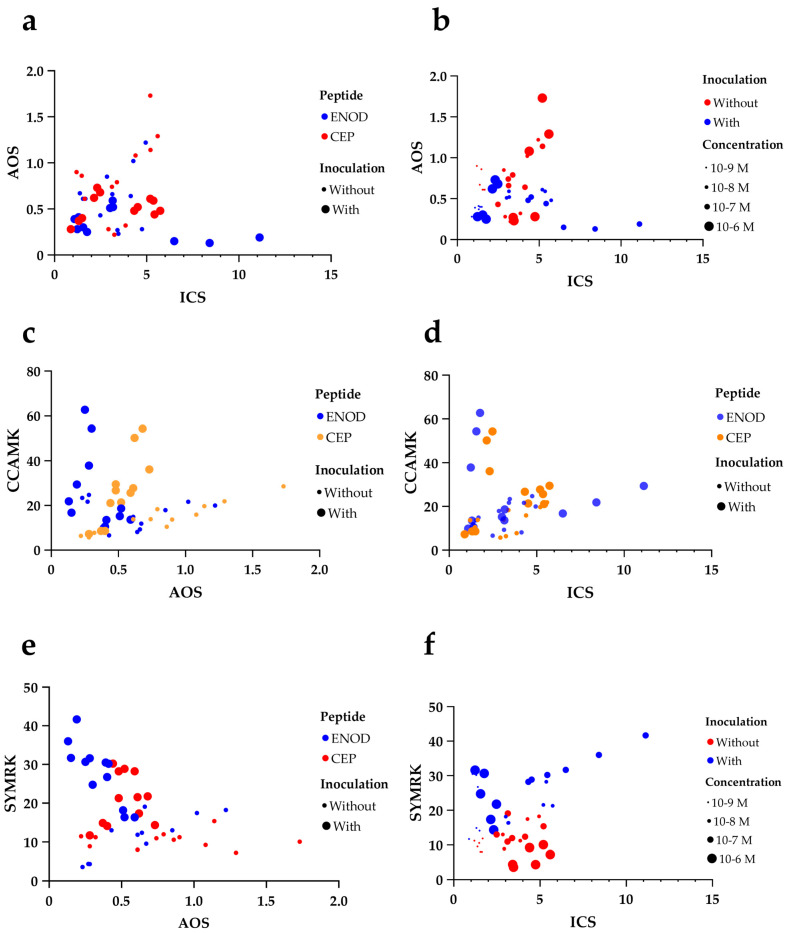
Multivariate bubble plot analysis of gene expression relationships between defense- and symbiotic-related genes in *Phaseolus vulgaris* under peptide and *Rhizobium* treatments: (**a**) *PvAOS* vs. *PvICS* colored by peptide type; (**b**) *PvAOS* vs. *PvICS* colored by inoculation and sized by concentration; (**c**) *PvCCaMK* vs. *PvAOS* colored by peptide type and inoculation; (**d**) *PvCCaMK* vs. *PvICS* colored by peptide type and inoculation; (**e**) *PvSYMRK* vs. *PvAOS* colored by peptide type and inoculation; (**f**) *PvSYMRK* vs. *PvICS* colored by inoculation and sized by concentration.

The expression of *PvAOS*, a key enzyme in the jasmonate biosynthetic pathway, was consistently downregulated across most peptide treatments, particularly in combination with *Rhizobium* (Figure 2a,b). The lowest expression was observed in the ENOD 10^−7^ M + *Rhizobium* treatment, which reduced *PvAOS* transcript levels by approximately 66% compared to the uninoculated control. CEP1 at the same concentration also decreased *AOS* expression by 44% (Figure 1f). The inhibitory effect was dose-dependent, with the strongest suppression occurring at intermediate peptide concentrations (10^−7^ to 10^−8^ M). This pattern suggests that peptide signaling may attenuate jasmonate-mediated immune responses, potentially facilitating symbiont colonization and nodule initiation by reducing plant immune barriers.

In contrast, the expression of *PvICS*, a marker of the salicylic acid pathway, showed more variable responses (Figure 2d,f). Some treatments resulted in slight upregulation, while others had no statistically significant effect. For instance, CEP1 at 10^−8^ M without *Rhizobium* led to a modest increase in *ICS* expression, whereas ENOD40 at the same concentration downregulated ICS expression (Figure 1g). The lack of a consistent trend indicates that the salicylate branch of plant defense may be less responsive to peptide-mediated modulation, or may be regulated independently of the symbiotic signals activated by ENOD40 and CEP1.

### 2.3. Interaction Between Peptide Type, Concentration, and Inoculation Reveals Synergistic Activation of Symbiotic Genes

The three-way ANOVA revealed that peptide concentration was the most significant factor across all genes, followed by inoculation with *Rhizobium* and the interaction between peptide type and concentration, indicating a complex modulation of gene expression by these factors. Beyond the main effects of each factor, the analysis revealed significant two-way and three-way interactions that highlight the complex regulatory dynamics between peptide signaling and microbial cues (Tables S2–S8).

The most notable interactions were observed in the expression of *PvENOD40*, *PvSYMRK*, and *PvCCaMK*. In all three cases, expression was not merely additive when combining peptides and *Rhizobium*, but rather synergistic, especially at intermediate concentrations, with the latter being the factor that most influenced the variation in ENOD expression. The concentration–inoculation interaction is particularly evident when comparing treatments with and without inoculation. The presence of *Rhizobium* enhances gene expression across all doses, especially at 10^−7^ M, where expression with inoculation is significantly higher than without, for both *ENOD40* and *CEP1*. This interaction suggests that the efficacy of the phytohormones in activating *ENOD* expression is modulated by the bacterial symbiosis.

Similar patterns were observed for *PvSYMRK* and *PvCCaMK*. The expression of *PvSYMRK* was significantly higher in inoculated plants compared with uninoculated plants, except in the ENOD40 10^−8^ M treatment, where inoculation was the most important factor (Table S3). In the case of *PvCCaMK*, both ENOD40 and CEP1 showed an upregulated expression with and without *Rhizobium*, with a slight increase in the inoculated treatments (Figure 1c). ANOVA shows concentration to be the most determinant factor in CCAMK gene expression, and two-way and three-way interactions are significant (Table S4).

Importantly, interaction effects varied between peptides. In the case of CYCLOPS, three-way interaction is the most determinant factor for its expression (Table S5), and in AOS, concentration × peptide interaction represents 38.9% of the total variation, being the most important factor in this gene (Table S7).

### 2.4. Correlation and Multivariate Analysis Reveal Co-Regulated Modules Among Symbiotic and Defense Genes

A comprehensive understanding of the transcriptional relationships among genes modulated by peptide and *Rhizobium* treatments was obtained through the integration of a Pearson correlation matrix and principal component analysis (PCA). This analysis revealed clear patterns of co-regulation and functional clustering, highlighting the distinct transcriptional dynamics between symbiosis-related and defense-related genes analyzed in this study.

**Figure 3:**
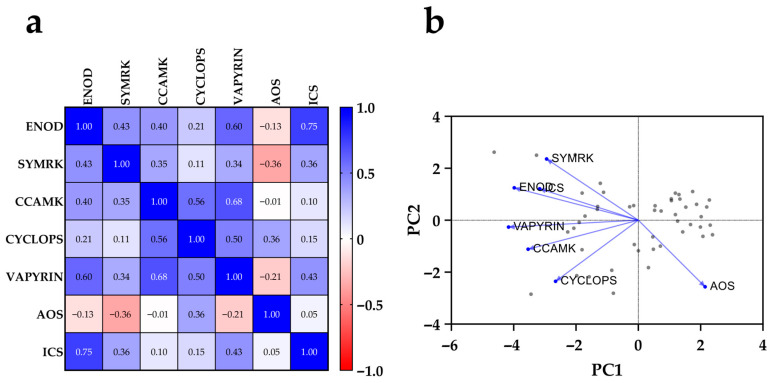
Correlation and principal component analysis of gene expression profiles in *Phaseolus vulgaris* reveals functional clustering of symbiotic and defense pathways: (**a**) Pearson correlation matrix among seven target genes; (**b**) principal component analysis (PCA) biplot of gene loadings and treatment scores.

As shown in the correlation matrix (Figure 3a), strong positive correlations were observed among several Sym pathway genes. Notably, *PvCYCLOPS* correlated strongly with *PvCCaMK* (r = 0.56) and *PvVAPYRIN* (r = 0.50), while *PvVAPYRIN* also showed a high correlation with *PvCCaMK* (r = 0.68). These relationships reflect their coordinated transcriptional regulation within the calcium-dependent signaling module of nodulation. *PvENOD40* was moderately correlated with *PvSYMRK* (r = 0.43), *PvCCaMK* (r = 0.40), and *PvVAPYRIN* (r = 0.60), consistent with its upstream regulatory role. In contrast, the correlations between symbiotic and defense genes were generally weak or negative. For example, *PvAOS* showed a mild negative correlation with *PvSYMRK* (r = –0.36) and *PvENOD40* (r = –0.13), suggesting a mutual antagonism between jasmonate-mediated defense and symbiotic gene expression.

Interestingly, *PvICS*, associated with salicylic acid biosynthesis, exhibited a strong positive correlation with *PvENOD40* (r = 0.75), and moderate correlations with other Sym genes, implying that salicylate signaling may not be antagonistic—and possibly even supportive—of symbiotic processes, in contrast to jasmonates. Additionally, *PvAOS* and *PvICS* were positively correlated (r = 0.05), indicating partial co-regulation of general immune responses, although their functional divergence is evident from expression patterns.

The PCA biplot (Figure 3b) further highlighted the separation of gene clusters based on their transcriptional behavior across treatments. PC1 explained the main variation driven by the antagonistic behavior of *PvAOS* relative to symbiotic genes. The positioning of *PvAOS* in the opposite quadrant from *PvSYMRK*, *PvCCaMK*, and *PvVAPYRIN* emphasizes the transcriptional decoupling between defense suppression and symbiotic activation. PC2 contributed to separating *PvENOD40*, *PvICS*, and *PvSYMRK*, suggesting distinct but overlapping regulatory inputs, possibly reflecting their upstream positions and broader signaling influence.

### 2.5. Foliar Application of Synthetic Peptides Enhances Biomass, Nodulation, and Yield Components Under Greenhouse Conditions

**Figure 4:**
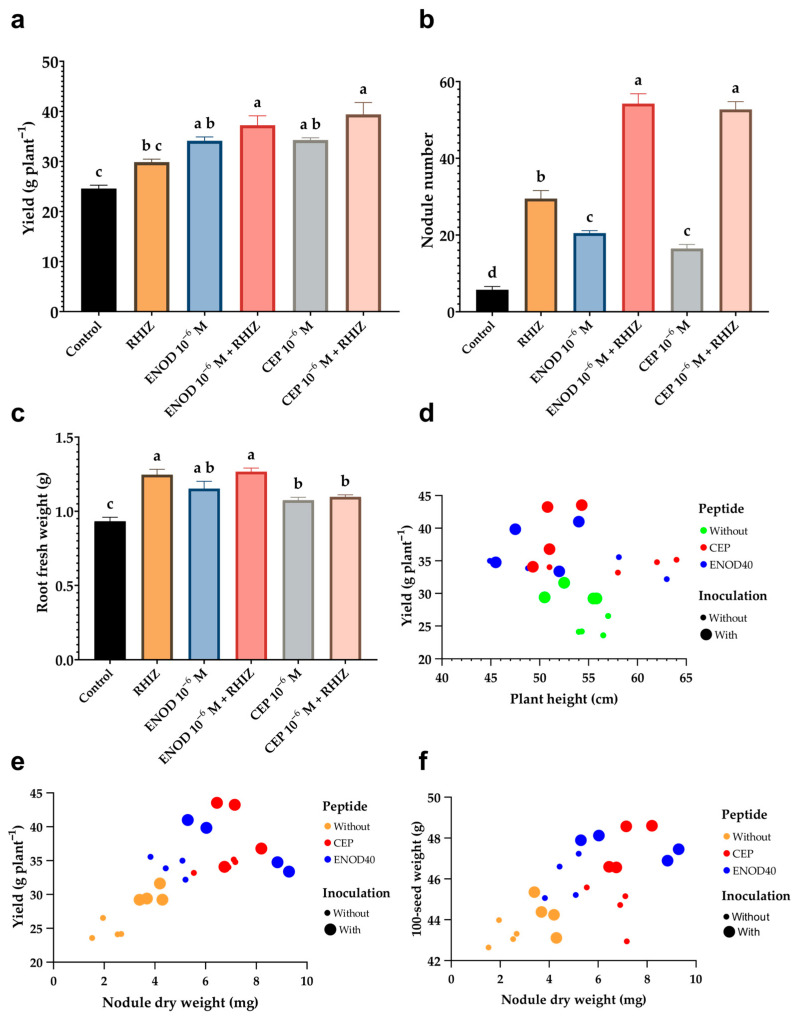
Greenhouse evaluation of morphological, symbiotic, and yield traits in *Phaseolus vulgaris* under foliar application of ENOD40 and CEP1 peptides and *Rhizobium tropici* inoculation: (**a**) grain yield per plant; (**b**) number of nodules per plant; (**c**) fresh root biomass; (**d**) correlation between plant height and yield; (**e**) correlation between nodule dry weight and grain yield; (**f**) correlation between 100-seed weight and nodule dry weight.

The greenhouse experiment provided evidence that foliar application of ENOD40 and CEP1 peptides, particularly in combination with *Rhizobium*, positively influenced vegetative growth, nodulation, and reproductive performance in common bean plants (Figure 4). Morphological variables showed differential responses across treatments. Although plant height did not differ significantly between treatments, other growth parameters were strongly affected (Table 1).

The inoculated treatment produced the highest fresh shoot biomass (59.16 g), followed closely by the ENOD 10^−6^ M + *Rhizobium* treatment with 58.28 g, and CEP 10^−6^ M + *Rhizobium* with 55.88 g. Dry root biomass was maximized in the ENOD 10^−6^ M + *Rhizobium* treatment (1.267 g), similar to the inoculated treatment without peptides, indicating that *Rhizobium* can enhance below-ground growth by itself. CEP and inoculation treatment had the highest root fresh weight (19.64 g), so ENOD and CEP-treated plants accumulated more dry shoot and root biomass compared to controls, reinforcing the stimulatory effect of synthetic peptides on vegetative development.

Nodulation parameters also responded significantly to peptide treatments. The number of nodules per plant increased substantially in all peptide-treated groups, particularly those co-inoculated with *Rhizobium*. The ENOD40 + *Rhizobium* treatment produced 54 nodules per plant, compared to only 5.75 nodules in the uninoculated control. In contrast, CEP1 and ENOD alone yielded lower nodules per plant compared to the inoculated control. Both peptides also enhanced fresh and dry nodule weights, indicating not only a greater number but also more developed nodules. These results demonstrate that ENOD40 and CEP1 act as nodulation-promoting agents, enhancing both the initiation and development of symbiotic structures when used in tandem with *Rhizobium* (Figure 4b,e,f).

In terms of reproductive performance, significant improvements were observed in pod and seed traits. The highest number of pods per plant was recorded in CEP + *Rhizobium* (17.82), followed closely by CEP1 alone (17.5). The number of seeds per pod ranged from 4.24 in the control to 4.745 in ENOD40 + *Rhizobium* plants, with modest but consistent increases in all peptide-based treatments. Pod length and diameter were also greater in peptide-treated plants, particularly under combined application with *Rhizobium*. Furthermore, the 100-seed weight increased from 43.25 g in the control to over 47.6 g in the peptide-treated groups, reflecting enhanced seed filling (Figure 4f). Total grain yield per plant was significantly boosted by both peptides: CEP1 + *Rhizobium* reached 39.4 g/plant, while ENOD40 + *Rhizobium* yielded 37.2 g/plant, compared to only 24.6 g/plant in the control (Figure 4a).

### 2.6. Field Application of ENOD40 and CEP1 Improves Nodulation and Increases Grain Yield in Common Bean

**Figure 5:**
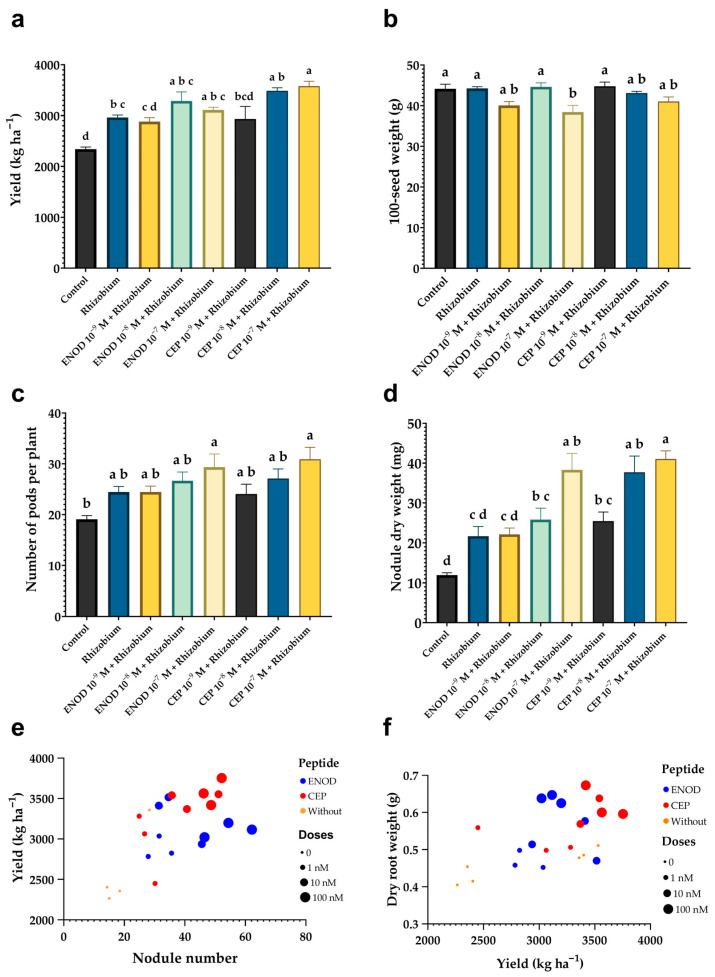
Field evaluation of grain yield, reproductive traits, and nodulation parameters in *Phaseolus vulgaris* treated with foliar ENOD40 and CEP1 peptides and *Rhizobium tropici*: (**a**) grain yield per hectare; (**b**) 100-seed weight; (**c**) number of pods per plant; (**d**) nodule dry weight per plant; (**e**) correlation between number of nodules and grain yield; (**f**) correlation between root dry biomass and grain yield.

The field experiment demonstrated that foliar application of ENOD40 and CEP1 peptides, in combination with *Rhizobium*, significantly improved plant performance under agronomic conditions. The best results were observed at intermediate peptide concentrations, particularly 10^−7^ M and 10^−8^ M (Figure 5). The same pattern as that observed in the greenhouse was seen in plant height in field, without significant differences in field, but significant increases were noted in biomass accumulation, nodulation parameters, and yield-related traits (Table 2).

**Figure 6:**
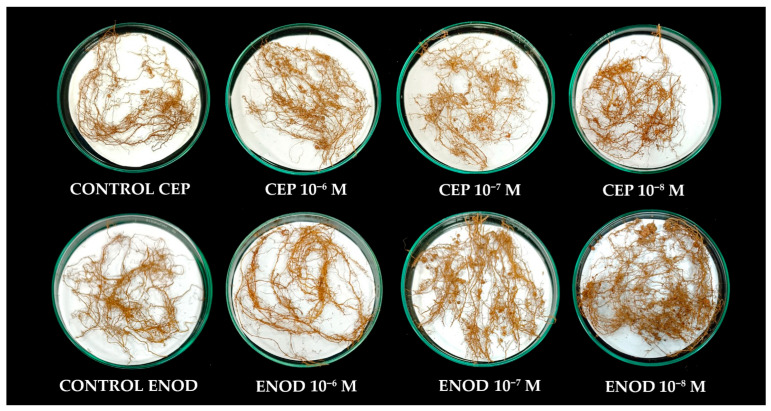
Visual comparison of root system architecture in early harvested field-grown common bean treated with foliar ENOD40 and CEP1 peptides at different concentrations (control, 10^−6^ M, 10^−7^ M, and 10^−8^ M).

Root nodulation responded positively to peptide treatments (Figure 6). The highest number of nodules per plant was recorded in the ENOD40 10^−7^ M + *Rhizobium* treatment (54.3 nodules), followed by CEP1 10^−7^ M + *Rhizobium* (49 nodules) and CEP1 10^−8^ M + *Rhizobium* (42.49 nodules), all of which exceeded the inoculated control (15.9 nodules). These increases were also reflected in fresh and dry weight of nodules, suggesting enhanced symbiotic efficiency. Notably, even at lower concentrations (10^−9^ M), peptide-treated plants exhibited higher nodule numbers than untreated controls, indicating that ENOD40 and CEP1 promote nodulation across a broad dose range.

Yield-related traits also showed marked improvement in response to peptide treatments. The highest grain yield was obtained with CEP1 at 10^−7^ M + *Rhizobium*, reaching 3578 kg ha^−1^, followed by CEP1 at 10^−8^ M + *Rhizobium* (3487 kg ha^−1^) and ENOD40 at 10^−8^ M + *Rhizobium* (3287 kg ha^−1^). These yields significantly exceeded those of the uninoculated control (2341 kg ha^−1^) and the *Rhizobium*-only treatment (2962 kg ha^−1^), highlighting the synergistic effect of peptides and microbial inoculation. Other components of yield also improved: pod width was highest in ENOD40 10^−9^ M + *Rhizobium* (1.16 cm), and ENOD 10^−8^ M + *Rhizobium* (1.15 cm) showed similar results, compared to only 0.96 cm in the control group (Table 2).

The number of pods per plant and seeds per pod were also positively influenced. Treatments with ENOD40 10^−7^ M and CEP1 10^−7^ M, both combined with *Rhizobium*, led to significantly more reproductive structures, contributing to higher overall productivity. Additionally, the 100-seed weight was higher in peptide-treated groups, indicating better seed filling and possibly improved nutrient mobilization during grain development, but not statistically different with respect to control.

Altogether, these results confirm that field application of ENOD40 and CEP1 peptides enhances nodulation capacity, reproductive development, and final yield in common bean. The observed effects were dose-dependent and more pronounced at 10^−7^ and 10^−8^ M, supporting the potential of these peptides as bioactive tools in sustainable agricultural systems.

## 3. Discussion

### 3.1. Synthetic Peptides as Modulators of Symbiotic Gene Expression in Common Bean

The application of the synthetic peptides ENOD40 and CEP1 has demonstrated a significant impact on the expression of key symbiotic genes in *Phaseolus vulgaris*: *ENOD40*, *SYMRK*, *CCaMK*, *CYCLOPS*, and *VAPYRIN*. These genes are integral components of the common symbiosis signaling pathway (CSSP), which orchestrates the establishment of nitrogen-fixing nodules in legumes [15,17]. The observed upregulation suggests that these peptides can effectively mimic endogenous signaling molecules [27,42], thereby enhancing the plant’s readiness for symbiotic interactions [43,44].

ENOD40, one of the earliest nodulin genes, is known to be induced by rhizobial Nod factors and cytokinin, playing a pivotal role in initiating the cortical cell divisions necessary for nodule formation [33,45,46,47]. Its expression precedes visible nodule development, indicating its function in the early stages of symbiosis [48,49,50]. Moreover, ENOD40 encodes small peptides that have been shown to bind to sucrose synthase, suggesting a role in regulating carbon allocation during nodule development [51].

Similarly, CEP1 peptides are part of a family of small, secreted peptides that regulate root architecture and nodulation in response to nitrogen availability [34,40,41,52,53]. In *Medicago truncatula*, CEP1 has been shown to modulate lateral root and nodule numbers, acting through a systemic signaling mechanism that involves the CEP receptor CEPR1 [52]. The application of synthetic CEP1 peptides in *P. vulgaris* likely engages similar pathways, enhancing the expression of symbiotic genes and promoting nodule formation [34,40].

The synergistic effect observed when combining peptide application with *Rhizobium* inoculation underscores the complex interplay between plant-derived signals and microbial factors [54,55]. This interaction likely amplifies the signaling cascade, leading to a more robust activation of symbiotic genes [56,57]. Such findings align with previous studies demonstrating that the integration of multiple signaling inputs is crucial for the successful establishment of legume–rhizobia symbiosis [24,58,59,60].

Furthermore, the upregulation of *PvSYMRK*, *PvCCaMK*, and *PvCYCLOPS* indicates that synthetic peptides can influence downstream components of the CSSP [16]. SYMRK, a receptor-like kinase, is essential for the perception of Nod factors and the initiation of calcium spiking, a key secondary messenger in symbiotic signaling [19,61,62]. CCaMK decodes calcium oscillations, activating CYCLOPS, a transcriptional activator that regulates nodule-specific gene expression [20,63,64,65,66]. The enhanced expression of these genes suggests that synthetic peptides can potentiate the entire signaling pathway, from perception to transcriptional response [67,68,69].

The observed increase in *PvVAPYRIN* expression further supports the role of synthetic peptides in facilitating symbiosis. VAPYRIN is involved in the intracellular accommodation of rhizobia, a critical step for the formation of functional nodules [23,70,71]. Its upregulation implies that peptide treatments not only initiate signaling cascades but also promote the structural adaptations necessary for symbiosis [72].

Future work should include other central symbiotic regulators such as NIN, which was not analyzed in this study but is known to act downstream of the CSSP and plays a key role in nodulation gene networks

### 3.2. Modulation of Defense-Related Gene Expression by Peptide Treatments

The application of the synthetic peptides ENOD40 and CEP1 in *Phaseolus vulgaris* not only enhanced the expression of symbiotic genes but also modulated the expression of defense-related genes, notably *PvAOS* and *PvICS*, which are associated with the jasmonic acid (JA) and salicylic acid (SA) signaling pathways, respectively. This modulation suggests a complex interplay between symbiotic signaling and plant defense mechanisms [73,74,75].

The observed downregulation of *PvAOS*, a key gene in the JA biosynthesis pathway, implies a suppression of JA-mediated defense responses (Figure 1). JA is known to negatively regulate nodulation by inhibiting early nodulin gene expression and infection thread formation [76,77]. For instance, exogenous application of JA has been shown to suppress the expression of early nodulin genes such as *ENOD11* and *RIP1*, thereby reducing nodule formation in legumes like *Lotus japonicus* [78,79]. Therefore, the downregulation of *PvAOS* by peptide treatments may alleviate the inhibitory effects of JA on nodulation, facilitating a more conducive environment for symbiotic interactions [55].

Conversely, the upregulation of *PvICS*, involved in SA biosynthesis, suggests an enhancement of SA-mediated responses (Figure 1). While SA is traditionally associated with defense against biotrophic pathogens, its role in nodulation is more nuanced [76,80]. Elevated SA levels have been linked to both promotion and inhibition of nodulation, depending on the context and concentration [78,80]. For example, moderate increases in SA can enhance nodulation efficiency, whereas excessive SA accumulation may hinder nodule formation [81]. The upregulation of *PvICS* in response to peptide treatments might reflect a fine-tuned modulation of SA levels, balancing defense readiness with symbiotic compatibility [55].

The differential regulation of the JA and SA pathways by the ENOD40 and CEP1 peptides underscores the complexity of hormonal crosstalk during nodulation [35,38]. It is well-established that the JA and SA signaling pathways can exhibit antagonistic interactions, where the activation of one pathway suppresses the other [78]. By downregulating JA-associated genes and upregulating SA-associated genes, peptide treatments may shift the hormonal balance in favor of symbiosis, reducing defense-related barriers to rhizobial infection (Figure 2).

Furthermore, the modulation of defense-related gene expression by these peptides may involve interactions with other hormonal pathways, such as ethylene and abscisic acid (ABA), which also influence nodulation and defense responses [74,78]. For instance, ethylene is known to inhibit nodulation by suppressing Nod factor signaling and infection thread formation, while ABA can modulate both defense and symbiotic pathways [76]. The precise mechanisms by which ENOD40 and CEP1 peptides interact with these hormonal networks remain to be elucidated [78]

### 3.3. Interactions Between Peptide Type, Concentration, and Rhizobial Inoculation

The three-way ANOVA analysis revealed a significant influence of interactions between peptide type (ENOD40 or CEP1), peptide concentration (10^−6^ to 10^−9^ M), and *Rhizobium* inoculation on the expression of symbiotic genes in *Phaseolus vulgaris*. These interactions underscore the complexity of the regulatory networks governing nodulation and highlight the importance of optimizing multiple factors to enhance symbiotic efficiency [25].

The ENOD40 and CEP1 peptides, while both promoting nodulation, operate through distinct pathways. The differential effects observed between these peptides at various concentrations suggest that they may be modulating different aspects of the nodulation process [54].

The concentration-dependent effects observed in this study align with previous findings that peptide signaling is highly sensitive to dosage. Although a strictly linear dose–response relationship was not observed across all genes, the expression patterns exhibited gene-specific sensitivity, often displaying bell-shaped or bimodal responses. This is consistent with prior studies on peptide signaling, in which optimal bioactivity was shown to occur at intermediate concentrations due to receptor saturation or desensitization [82]. For instance, overexpression of MtCEP1 in *Medicago truncatula* led to increased nodule numbers, while higher concentrations of synthetic CEP1 peptides inhibited lateral root formation [82]. Similarly, the application of synthetic ENOD40 peptides has been shown to influence root architecture and nodule development in a concentration-dependent manner [35]. These findings highlight the necessity of precise peptide concentration management to achieve the desired outcomes in nodulation (Tables S2–S8).

The synergistic effects observed when combining peptide treatments with *Rhizobium* inoculation suggest that these peptides may prime the plant for symbiotic interactions, enhancing the plant’s responsiveness to rhizobial signals. This priming effect could involve the upregulation of Nod factor receptors or other components of the symbiotic signaling pathway, thereby facilitating more efficient nodule formation [60]. Such interactions underscore the potential of integrating peptide treatments with microbial inoculants to improve legume productivity [83].

### 3.4. Greenhouse Evaluation: Morphological, Symbiotic, and Agronomic Responses to Peptide Treatments

The improvements observed in vegetative biomass, nodulation, and grain yield parameters, especially when peptides were combined with *Rhizobium* inoculation, reflect a coordinated physiological response that integrates enhanced symbiotic signaling with metabolic support for growth and reproduction. These results are consistent with earlier studies reporting that the activation of the symbiotic pathway not only facilitates nitrogen acquisition but also influences plant architecture, biomass accumulation, and reproductive output [84,85].

Morphological improvements in shoot and root biomass in peptide-treated plants likely reflect early transcriptional priming of symbiotic genes, as discussed previously, leading to more efficient nitrogen assimilation and carbon allocation. This is aligned with the hypothesis that peptide signaling is capable of modulating broader growth-regulatory networks. Previous research under greenhouse conditions has demonstrated that enhanced nodulation correlates with improved photosynthetic capacity and shoot growth in common bean, particularly when nodulation is established early and efficiently [86,87]. In our case, ENOD40 and CEP1, especially when co-applied with *Rhizobium*, appear to accelerate this process, enhancing both vegetative development and symbiotic integration.

The increase in nodule number and biomass observed in this study mirrors findings by Nova-Franco et al. [88], who showed that the overexpression of ENOD40 homologs in legumes led to increased nodule primordia formation. Our results reinforce this outcome using exogenous peptide application rather than genetic manipulation, offering a more practical route for agronomic deployment [89]. Similarly, CEP1 has been shown to promote nodule formation through a systemic shoot-to-root signaling loop in *Medicago truncatula* [52], and our results suggest this mechanism may also be conserved in *P. vulgaris* when peptides are applied foliarly.

Yield improvements, especially in pod number, seed weight, and total grain mass per plant, are particularly noteworthy. These results confirm that nodulation benefits induced by peptide treatments translate into measurable agronomic gains, even under non-stressful greenhouse conditions. This is consistent with findings by Graham and Rosas [90] and later by Vargas et al. [91], who reported that increased nodulation and biological nitrogen fixation in greenhouse-grown beans led to significantly higher seed yields when nitrogen supply was limited. The peptide treatments thus appear to act as biostimulants, enhancing the efficiency of nitrogen assimilation and its partitioning toward reproductive development.

Moreover, the synergistic interaction between peptides and *Rhizobium* is key. While peptide-only treatments improved several parameters, the combination with microbial inoculation consistently yielded superior results. This reinforces the notion that peptide signaling does not replace the need for microbial interaction but rather enhances the plant’s sensitivity and preparedness for symbiosis. This concept of molecular priming has been described in legumes preconditioned by flavonoids or Nod factors [92], and our results suggest that ENOD40 and CEP1 may function similarly when delivered exogenously.

### 3.5. Field Evaluation: Morphological, Symbiotic, and Agronomic Responses to Peptide Treatments

The treatments that combined peptides at concentrations of 10^−7^ M and 10^−8^ M with *Rhizobium* inoculation produced the highest yield values, highlighting a synergistic interaction between exogenous peptide signals and microbial symbionts. These results support the hypothesis that synthetic peptides can modulate physiological and developmental processes in legumes even under the variable environmental conditions typical of field cultivation.

The increased nodulation observed in the field treatments echoes patterns previously reported in controlled environments. For instance, studies by Nova-Franco et al. [88] demonstrated that overexpression of ENOD40 in legumes increases nodule formation through modulation of cortical cell divisions and root cell fate. Our findings provide field-level validation of this concept, showing that ENOD40—when applied exogenously—can enhance nodulation efficiency beyond the basal levels induced by rhizobial inoculation alone. Similarly, CEP1 has been shown to function systemically to regulate nodule number in *Medicago truncatula* via shoot-derived signaling [93], and its effect observed in *P. vulgaris* suggests functional conservation across species.

Notably, the nodulation response observed in the field was not only quantitative (i.e., more nodules per plant), but also qualitative, as indicated by increases in nodule biomass. This is critical for effective nitrogen fixation, since larger and better-developed nodules tend to support more efficient symbiotic activity [84]. The consistency of nodulation enhancement across greenhouse and field trials indicates that peptide treatments remain effective even under non-controlled environments, making them promising candidates for agronomic use.

Yield-related traits responded markedly to peptide treatments. Grain yield in the CEP1 10^−7^ M + *Rhizobium* treatment exceeded 3300 kg ha^−1^, outperforming both the control and the *Rhizobium*-only inoculated plants. These increases were accompanied by improvements in pod number, pod diameter, seed weight, and seed number per pod—traits that together determine final productivity. Similar findings were reported by Vargas et al. [91] and later by Cántaro-Segura [94], who showed that improved nodulation and nitrogen fixation translate directly into greater reproductive investment and yield in beans, particularly under nitrogen-deficient conditions.

Importantly, the differential dose response observed in the field reinforces the concept that synthetic peptides act through concentration-dependent mechanisms. Lower concentrations such as 10^−9^ M were less effective, while intermediate doses (10^−7^ and 10^−8^ M) consistently triggered optimal physiological responses. This pattern aligns with reports from studies applying CEP peptides in *Medicago truncatula* and *Lotus japonicus*, where both root architecture and nodule numbers were strongly dependent on precise peptide dosage [52,95].

Environmental variability in the field often reduces the efficacy of biostimulant treatments observed in controlled environments. However, the maintenance of significant effects under field conditions—especially in nodulation and yield—demonstrates the robustness of the peptide treatments. The inclusion of a surfactant and adherent in the foliar application likely enhanced the uptake and systemic movement of the peptides, contributing to the observed outcomes. Furthermore, the early application of peptides (at 7 and 14 DAS) coincides with the critical window of nodulation, aligning with the optimal timing described by Ferguson et al. [96] for effective symbiotic establishment.

## 4. Materials and Methods

This research work was carried out in Laboratorio de Ecología Microbiana y Biotecnología (LEMYB), Department of Biology, Faculty of Sciences, UNALM.

### 4.1. Plant Material and Experimental Conditions

Seeds of *Phaseolus vulgaris* L. cultivar ‘Canario Centenario’, a variety developed by the Legumes and Oilseed Research Program from the Faculty of Agronomy, UNALM, were surface-sterilized using 70% ethanol for 3 min followed by 3% sodium hypochlorite for 3 min and thoroughly rinsed with sterile distilled water. Germinated seedlings were transferred to sterile 1.5 L pots containing an autoclaved substrate composed of a 1:1 (*v*/*v*) mixture of coarse and vermiculite. Plants were maintained in a growth chamber under controlled conditions (16 h photoperiod, 26 ± 2 °C, 70% relative humidity) until the application of treatments. Plants were watered with nitrogen-free Broughton and Dilworth solution [97].

### 4.2. Peptide Hormones and Treatments

Two synthetic peptide hormones, ENOD40 and CEP1, were selected based on their known regulatory functions in early nodulation and nitrogen signaling in legumes. The ENOD40 peptide was designed using the functional motif derived from the *Glycine max* precursor gene, while the CEP1 peptide was based on the conserved C-terminal region of the *Arabidopsis thaliana* CEP1 peptide, previously identified as a key regulator of systemic nitrogen deficiency signaling. The sequences used for synthesis were Met-Glu-Leu-Cys-Trp-Leu-Thr-Thr-Ile-His-Gly-Ser for *GmENOD40-1* [33], and Asp-Phe-Arg-Hyp-Thr-Asn-Hyp-Gly-Asn-Ser-Hyp-Gly-Val-Gly-His for *AtCEP1* [40]. Both peptides were synthesized by Pepmic Co., Ltd. (Suzhou, China). The products were purified to a minimum of 90% purity by high-performance liquid chromatography (HPLC), lyophilized, and stored at −20 °C until use.

To prepare the peptide solutions, each lyophilized peptide was initially dissolved in 100% dimethyl sulfoxide (DMSO) to create 1 mM stock solutions. These stocks were aliquoted and stored at −20 °C to prevent degradation through freeze–thaw cycles. Working solutions were freshly prepared for each application by diluting the stock solution in sterile deionized water to final concentrations of 10^−6^ M, 10^−7^ M, 10^−8^ M, and 10^−9^ M. In all working solutions, the final DMSO concentration was kept below 0.1% (*v*/*v*) to avoid any potential phytotoxicity.

For foliar application, each peptide solution was supplemented with an agricultural-grade non-ionic surfactant and adherent to ensure uniform spreading and leaf retention. Treatments were applied once, at the seedling stage, specifically at seven days after emergence, using a manual handheld sprayer to thoroughly cover the adaxial and abaxial leaf surfaces without runoff. Each plant received the treatment until full wetting of foliage was observed.

Each treatment was evaluated under two conditions: in the presence and absence of *Rhizobium tropici* CIAT899 [98]. The CIAT899 was from the laboratory’s strain bank (LEMYB), and was applied as a live inoculum at a concentration of 10^8^ CFU mL^−1^ at the sowing stage. This resulted in a full factorial design 2 × 4 × 2 encompassing peptide type, concentration, and microbial inoculation respectively. All treatments were applied under aseptic conditions using autoclaved substrates and sterile materials to prevent microbial contamination.

### 4.3. Sample Collection and RNA Extraction

Fresh roots were harvested three days after the treatment application, immediately frozen in liquid nitrogen, and stored at −80 °C. For RNA isolation, 100 mg of frozen root tissue was ground in liquid nitrogen and total RNA was extracted using the PureLink™ RNA Plant Reagent (Invitrogen, Waltham, MA, USA), following the manufacturer’s protocol. Residual genomic DNA was eliminated using the TURBO DNA-free™ Kit (Invitrogen, Waltham, MA, USA). RNA quality and integrity were assessed by agarose gel electrophoresis (1.5%) and quantified with a NanoDrop™ 2000 spectrophotometer (Thermo Fisher Scientific, Waltham, MA, USA).

### 4.4. cDNA Synthesis and Quantitative Real-Time PCR

First-strand cDNA was synthesized from 1 µg of total RNA using the High-Capacity cDNA Reverse Transcription Kit with RNase Inhibitor (Applied Biosystems, Waltham, MA, USA), following the manufacturer’s instructions. Quantitative real-time PCR (qPCR) was carried out using a QuantStudio™ 3 Real-Time PCR System (Applied Biosystems, Waltham, MA, USA) and PowerUp™ SYBR™ Green Master Mix (Applied Biosystems, Waltham, MA, USA). Each 10 µL reaction contained 5 µL of master mix, 0.2 µL of each primer (10 µM), 1 µL of diluted cDNA (1:10), and 3.6 µL of nuclease-free water. The amplification protocol included an initial denaturation step at 95 °C for 2 min, followed by 40 cycles of 95 °C for 15 s, 60 °C for 15 s, and 72 °C for 1 min). A melting curve program (95 °C for 15 s, 60 °C for 1 min, and 95 °C for 15 s) was performed to confirm specificity. Gene-specific primers were used to amplify *PvENOD40*, *PvSYMRK*, *PvCCaMK*, *PvCYCLOPS*, *PvVAPYRIN*, *PvAOS*, and *PvICS*. Primer sequences were designed using *P. vulgaris* genome data and previously published works described and verified for specificity (Table S1). *PvActin* was used as the internal reference gene. Relative gene expression was calculated using the 2^−ΔΔCt^ method.

### 4.5. Greenhouse Assay

Plants of *Phaseolus vulgaris* L. cv. ‘Canario Centenario’ were grown individually in 4 L plastic pots filled with a sterilized 1:1 mixture of coarse sand and vermiculite. The experiment was conducted under natural light. Six treatments were evaluated, consisting of the peptides at a fixed concentration of 10^−6^ M, applied alone or in combination with *Rhizobium tropici* CIAT 899, alongside uninoculated and inoculated controls. The treatments were as follows: T1—uninoculated control, T2—inoculated control, T3—ENOD40 10^−6^ M without *Rhizobium*, T4—ENOD40 10^−6^ M with *Rhizobium*, T5—CEP1 10^−6^ M without *Rhizobium*, and T6—CEP1 10^−6^ M with *Rhizobium*. Each treatment was applied twice as a foliar spray at 7 days and 14 days after plant emergence, using peptide solutions prepared at the target concentration and supplemented with non-ionic surfactant and adherent to facilitate uniform coverage. *Rhizobium* was applied by seed inoculation prior to sowing at a density of 10^8^ CFU mL^−1^. Irrigation was performed every other day with nitrogen-deficient Hoagland nutrient solution, adjusted to field capacity.

At flowering and physiological maturity (approximately 90 days after sowing), plants were harvested and evaluated. Morphological variables included plant height and fresh and dry root biomass. Symbiotic parameters included the number of nodules per plant and fresh and dry weight of nodules. Agronomic traits were assessed based on number of pods per plant, number of seeds per pod, 100-seed weight, pod length and diameter, and final grain yield per plant. All measurements were conducted on six plants per treatment, using standardized protocols for sampling, weighing and drying at 65 °C (or 50 °C for nodules).

### 4.6. Field Assay

The field trial was conducted in an experimental UNALM station located in La Molina, Lima, Peru (12°05′ S, 76°57′ W, 240 masl), characterized by an arid coastal climate. The soil at the site was classified as a sandy clay loam with good drainage and moderate water-holding capacity. According to the physicochemical analysis, the soil had a pH of 7.71 (slightly alkaline), electrical conductivity of 2.22 dS/m (slightly saline), and adequate organic matter content of 2.12%, with high levels of available phosphorus (39 ppm) and potassium (470 ppm). The experiment was arranged in a completely randomized block design with three replicates per treatment. Each experimental unit consisted of a 9.6 m^2^ plot, and plants were sown at a spacing of 30 cm between plants and 80 cm between rows. Eight treatments were evaluated: T1—uninoculated control, T2—inoculated control (*Rhizobium*), T3—CEP1 10^−9^ M + *Rhizobium*, T4—CEP1 10^−8^ M + *Rhizobium*, T5—CEP1 10^−7^ M + *Rhizobium*, T6—ENOD40 10^−9^ M + *Rhizobium*, T7—ENOD40 10^−8^ M + *Rhizobium*, and T8—ENOD40 10^−7^ M + *Rhizobium*.

Peptide solutions were prepared by dissolving the lyophilized peptides in dimethyl sulfoxide (DMSO) and diluting to the target concentrations with sterile water. An agricultural-grade non-ionic surfactant and adherent were added to ensure foliar adhesion. Peptides were applied by foliar spraying at 7 and 14 days after sowing using a manual backpack sprayer, ensuring complete wetting of foliage without runoff. Each treatment was applied early in the morning under calm weather conditions to maximize absorption and reduce volatilization losses. All seeds in the inoculated treatments had previously been coated with *Rhizobium* at a density of 10^8^ CFU mL^−1^. A basal fertilizer dose of 40-40-80 (N-P-K) was applied at planting based on the soil analysis recommendations, and weekly irrigations were performed.

At flowering and physiological maturity, morphological, symbiotic, and yield-related parameters were recorded. Morphological variables included plant height and fresh and dry biomass of shoot and root tissues. Symbiotic measurements included the number of nodules per plant, fresh and dry weight of nodules, and nodule size. Yield-related traits included number of pods per plant, seeds per pod, 100-seed weight, pod length and diameter, and total grain yield per plant. Data were collected from five randomly selected plants per plot to ensure representation. All measurements were performed using standardized agronomic protocols and analyzed statistically as described below.

### 4.7. Statistical Analysis

A three-way ANOVA was conducted to assess the effects of peptide type (ENOD40 or CEP1), peptide concentration (10^−6^ to 10^−9^ M), and *Rhizobium* inoculation (with or without) on the relative expression of seven target genes. Tukey’s HSD test was used for post hoc comparisons. A one-way ANOVA and Tukey’s HSD test were conducted for the greenhouse and field assays to compare treatments. All statistical analyses were performed in R version 4.3.1, and gene expression graphs were generated using GraphPad Prism 10.4.

## 5. Conclusions

This study provides the first evidence that the foliar application of the synthetic peptides ENOD40 and CEP1 can effectively enhance symbiotic signaling, nodulation, and yield in common bean (*Phaseolus vulgaris*) under both greenhouse and field conditions. By activating key symbiotic genes and modulating defense-related pathways, these peptides improved plant growth and reproductive performance in a concentration-dependent manner, with the greatest benefits observed when combined with *Rhizobium* inoculation. The consistent enhancement of nodulation and grain yield across environments highlights the potential of ENOD40 and CEP1 as functional biostimulants for legume crops. It supports their integration into sustainable practices aimed at improving biological nitrogen fixation and reducing dependence on synthetic nitrogen inputs. While promising, this work has limitations: nitrogen fixation and plant N status were not quantified directly (e.g., ARA or ^15^N), peptide uptake/stability and receptor engagement were not measured, gene expression was profiled at a single early time point using one reference gene, and experiments involved a single cultivar and site. Future efforts should pair peptide treatments with direct nitrogenase and plant-N assays, JA/SA profiling, receptor- and pathway-level validation, optimization of dose–timing/formulation, and multi-environment testing across cultivars and soils to consolidate agronomic recommendations.

## Figures and Tables

**Table 1:** Morphological, nodulation, and yield traits of common bean plants grown under greenhouse conditions.

Treatments	Plant Height		Fresh Foliage Weight (g)		Fresh Root Weight (g)		Fresh Nodule Weight (mg)		Dry Nodule Weight (mg)		Dry Foliage Weight (g)		Dry Root Weight (g)		Nodule Number		Number of Pods per Plant		Number of Seeds per Pod		100-Seed Weight (g)		Pod Length (cm)		Pod Width (cm)		Yield (g/Plant)	
Control	55.45	a	47.81	c	7.718	c	14.45	d	2.168	d	6.655	c	0.932	c	5.75	d	13.41	b	4.24	c	43.25	c	11.52	a	1.105	bc	24.58	c
+*Rhizobium*	53.58	a	59.16	a	14.02	b	25.94	cd	3.892	cd	8.576	a	1.246	a	29.5	b	15.54	ab	4.348	bc	44.27	bc	11.29	a	1.088	c	29.86	bc
ENOD 10^−6^ M	53.7	a	55.1	abc	17.01	ab	30.9	bc	4.635	bc	7.965	ab	1.153	ab	20.5	c	16.34	a	4.545	ab	46.03	ab	11.17	a	1.1	bc	34.14	ab
ENOD 10^−6^ M + *Rhizobium*	49.75	a	58.28	ab	18.25	ab	49.08	a	7.362	a	8.589	a	1.267	a	54.25	a	16.49	a	4.745	a	47.59	a	11.35	a	1.19	abc	37.24	a
CEP 10^−6^ M	58.75	a	49.11	bc	16.1	ab	44.53	ab	6.679	ab	7.273	bc	1.075	b	16.5	c	17.5	a	4.398	bc	44.6	bc	11.73	a	1.235	ab	34.28	ab
CEP 10^−6^ M + *Rhizobium*	51.35	a	55.88	abc	19.64	a	47.56	a	7.133	a	7.825	ab	1.097	b	52.75	a	17.82	a	4.645	ab	47.58	a	11.6	a	1.265	a	39.41	a
*p* value	0.1584		0.007		<0.0001		<0.0001		<0.0001		<0.0001		<0.0001		<0.0001		0.0008		0.0004		<0.0001		0.0852		0.0017		<0.0001	
Significance	ns		**		****		****		****		****		****		****		***		***		****		ns		**		****	

Data represent means ± SME, with different letters indicating significant differences (*p* < 0.05, Tukey’s HSD test). *p*-value: ns: insignificant; **: *p* < 0.01; ***: *p* < 0.001; ****: *p* < 0.0001

**Table 2:** Morphological, nodulation, and yield traits of field-grown beans.

**Treatment**	Plant Height (cm)		Fresh Foliage Weight (g)		Fresh Root Weight (g)		Fresh Nodule Weight (mg)		Dry Nodule Weight (mg)		Dry Foliage Weight (g)		Dry Root Weight (g)		Nodule Number		Nodule Size (mm)		Number of Pods per Plant		Number of Seeds per Pod		100-Seed Weight (g)		Pod Length (cm)		Pod Width (cm)		Yield (kg ha^−1^)	
Control	51.27	c	93.97	c	2.951	c	82.49	d	11.88	d	13.84	b	0.4247	d	15.93	d	2.98	c	19.06	b	3.583	c	44.13	a	12.05	a	0.964	c	2341	d
+*Rhizobium*	55.5	ab	148.3	abc	3.363	bc	150.3	cd	21.67	cd	21.8	ab	0.4913	cd	32.53	c	3.15	bc	24.44	ab	4.542	a	44.24	a	11.77	a	1.051	bc	2962	abc
ENOD 10^−9^ M + *Rhizobium* + NPK	52.32	bc	114.4	bc	3.247	bc	152.5	cd	22.13	cd	16.59	b	0.4693	cd	31.67	c	4.36	abc	24.46	ab	4.184	ab	40.07	ab	12.01	a	1.164	a	2880	cd
ENOD 10^−8^ M + *Rhizobium* + NPK	51.37	c	97.49	c	3.523	bc	177.6	bc	25.83	bc	14.35	b	0.5203	bc	37.2	bc	4.59	abc	26.66	ab	3.959	abc	44.64	a	12.39	a	1.151	ab	3287	abc
ENOD 10^−7^ M + *Rhizobium* + NPK	57.44	a	183.5	a	4.357	a	263.2	ab	38.32	ab	26.8	a	0.6367	a	54.35	a	4.923	ab	29.33	a	3.867	bc	38.42	b	11.88	a	1.087	ab	3111	abc
CEP 10^−9^ M + *Rhizobium* + NPK	51.82	bc	121.3	abc	3.563	b	174	bc	25.43	bc	17.79	ab	0.521	bc	27.25	cd	3.857	abc	24.06	ab	3.888	bc	44.75	a	11.28	a	1.053	bc	2931	bcd
CEP 10^−8^ M + *Rhizobium* + NPK	55.45	ab	143.9	abc	4.197	a	255.5	ab	37.69	ab	21.37	ab	0.6027	ab	42.49	abc	4.713	abc	27.1	ab	4.257	ab	43.1	ab	12.27	a	1.146	ab	3487	ab
CEP 10^−7^ M + *Rhizobium* + NPK	59.36	a	176.1	ab	4.263	a	286	a	41.02	a	26.07	a	0.623	a	49.02	ab	5.497	a	30.88	a	4.039	abc	41.04	ab	11.28	a	1.075	ab	3578	a

Data represent means ± SME, with different letters indicating significant differences (*p* < 0.05, Tukey’s HSD test).

## Data Availability

The original contributions presented in this study are included in the article/Supplementary Material. Further inquiries can be directed to the corresponding authors.

## Supplementary Materials

The following supporting information can be downloaded at: https://www.mdpi.com/article/10.3390/plants14172786/s1, Table S1: Primer information, Table S2: Three-way ANOVA for ENOD gene expression analysis, Table S3: Three-way ANOVA for SYMRK gene expression analysis, Table S4: Three-way ANOVA for CCAMK gene expression analysis, Table S5: Three-way ANOVA for CYCLOPS gene expression analysis, Table S6: Three-way ANOVA for VAPYRIN gene expression analysis, Table S7: Three-way ANOVA for AOS gene expression analysis, Table S8: Three-way ANOVA for ICS1 gene expression analysis, Figure S1: Relative expression of ENOD gene in *Phaseolus vulgaris* roots in response to different concentrations of ENOD40 and CEP1 peptides with and without *Rhizobium* inoculation, Figure S2: Relative expression of SYMRK gene in *Phaseolus vulgaris* roots in response to different concentrations of ENOD40 and CEP1 peptides with and without *Rhizobium* inoculation, Figure S3: Relative expression of CCAMK gene in *Phaseolus vulgaris* roots in response to different concentrations of ENOD40 and CEP1 peptides with and without *Rhizobium* inoculation, Figure S4: Relative expression of CYCLOPS gene in *Phaseolus vulgaris* roots in response to different concentrations of ENOD40 and CEP1 peptides with and without *Rhizobium* inoculation, Figure S5: Relative expression of VAPYRIN gene in *Phaseolus vulgaris* roots in response to different concentrations of ENOD40 and CEP1 peptides with and without *Rhizobium* inoculation, Figure S6: Relative expression of AOS gene in *Phaseolus vulgaris* roots in response to different concentrations of ENOD40 and CEP1 peptides with and without *Rhizobium* inoculation, Figure S7: Relative expression of ICS1 gene in *Phaseolus vulgaris* roots in response to different concentrations of ENOD40 and CEP1 peptides with and without *Rhizobium* inoculation, Figure S8: Common bean plant root sample treated with ENOD 10^−7^ M and inoculated with *Rhizobium*, Figure S9: Common bean plant root harvested from the field with *Rhizobium* nodules.

## References

[1] Stagnari F., Maggio A., Galieni A., Pisante M. (2017). Multiple Benefits of Legumes for Agriculture Sustainability: An Overview. Chem. Biol. Technol. Agric..

[2] Wang T., Balla B., Kovács S., Kereszt A. (2022). Varietas Delectat: Exploring Natural Variations in Nitrogen-Fixing Symbiosis Research. Front. Plant Sci..

[3] Flores-Duarte N.J., Mateos-Naranjo E., Redondo-Gómez S., Pajuelo E., Rodriguez-Llorente I.D., Navarro-Torre S. (2022). Role of Nodulation-Enhancing Rhizobacteria in the Promotion of Medicago Sativa Development in Nutrient-Poor Soils. Plants.

[4] Parniske M. (2008). Arbuscular Mycorrhiza: The Mother of Plant Root Endosymbioses. Nat. Rev. Microbiol..

[5] Albrecht C., Geurts R., Bisseling T. (1999). Legume Nodulation and Mycorrhizae Formation; Two Extremes in Host Specificity Meet. EMBO J..

[6] Mukherjee A., Ané J. (2011). Plant Hormones and Initiation of Legume Nodulation and Arbuscular Mycorrhization. Ecological Aspects of Nitrogen Metabolism in Plants.

[7] Mohanta T.K., Bae H. (2015). Functional Genomics and Signaling Events in Mycorrhizal Symbiosis. J. Plant Interact..

[8] Rathna Priya T.S., Manickavasagan A. (2020). Common Bean. Pulses.

[9] Blair M.W., Soler A., Cortés A.J. (2012). Diversification and Population Structure in Common Beans (*Phaseolus vulgaris* L.). PLoS ONE.

[10] Razafintsalama H., Trap J., Rabary B., Razakatiana A.T.E., Ramanankierana H., Rabeharisoa L., Becquer T. (2022). Effect of *Rhizobium* Inoculation on Growth of Common Bean in Low-Fertility Tropical Soil Amended with Phosphorus and Lime. Sustainability.

[11] Karavidas I., Ntatsi G., Vougeleka V., Karkanis A., Ntanasi T., Saitanis C., Agathokleous E., Ropokis A., Sabatino L., Tran F. (2022). Agronomic Practices to Increase the Yield and Quality of Common Bean (*Phaseolus vulgaris* L.): A Systematic Review. Agronomy.

[12] Horácio E.H., Zambrano Gavilanes F.E., Feliciano M.V., de Moraes J.G., Zucareli C., Andrade D.S., Maddela N.R., Prasad R. (2024). Exploring the Interaction Effects between Common Bean Cultivars and Rhizobia Inoculation on Plant Growth and Yield. J. Agric. Food Res..

[13] Korir H., Mungai N.W., Thuita M., Hamba Y., Masso C. (2017). Co-Inoculation Effect of Rhizobia and Plant Growth Promoting Rhizobacteria on Common Bean Growth in a Low Phosphorus Soil. Front. Plant Sci..

[14] Desbrosses G.J., Stougaard J. (2011). Root Nodulation: A Paradigm for How Plant-Microbe Symbiosis Influences Host Developmental Pathways. Cell Host Microbe.

[15] De Bruijn F.J. (2020). The Common Symbiotic Signaling Pathway (CSSP or SYM). The Model Legume Medicago truncatula.

[16] Kumar N., Srivastava P., Vishwakarma K., Kumar R., Kuppala H., Maheshwari S.K., Vats S., Varma A., Tripathi S., Prasad R. (2020). The *Rhizobium*–Plant Symbiosis: State of the Art. Plant Microbe Symbiosis.

[17] Radhakrishnan G.V., Keller J., Rich M.K., Vernié T., Mbadinga Mbadinga D.L., Vigneron N., Cottret L., Clemente H.S., Libourel C., Cheema J. (2020). An Ancestral Signalling Pathway Is Conserved in Intracellular Symbioses-Forming Plant Lineages. Nat. Plants USA.

[18] Gherbi H., Markmann K., Svistoonoff S., Estevan J., Autran D., Giczey G., Auguy F., Péret B., Laplaze L., Franche C. (2008). SymRK Defines a Common Genetic Basis for Plant Root Endosymbioses with Arbuscular Mycorrhiza Fungi, Rhizobia, and Frankia Bacteria. Proc. Natl. Acad. Sci. USA.

[19] Holsters M. (2008). SYMRK, an Enigmatic Receptor Guarding and Guiding Microbial Endosymbioses with Plant Roots. Proc. Natl. Acad. Sci. USA.

[20] Gong X., Jensen E., Bucerius S., Parniske M. (2022). A CCaMK/Cyclops Response Element in the Promoter of Lotus Japonicus Calcium-Binding Protein 1 (CBP1) Mediates Transcriptional Activation in Root Symbioses. New Phytol..

[21] Limpens E., Bisseling T. (2014). CYCLOPS: A New Vision on *Rhizobium*-Induced Nodule Organogenesis. Cell Host Microbe.

[22] Capoen W., Oldroyd G. (2008). How CYCLOPS Keeps an Eye on Plant Symbiosis. Proc. Natl. Acad. Sci. USA.

[23] Murray J.D., Muni R.R.D., Torres-Jerez I., Tang Y., Allen S., Andriankaja M., Li G., Laxmi A., Cheng X., Wen J. (2011). Vapyrin, a Gene Essential for Intracellular Progression of Arbuscular Mycorrhizal Symbiosis, Is Also Essential for Infection by Rhizobia in the Nodule Symbiosis of Medicago Truncatula. Plant J..

[24] Shumilina J., Soboleva A., Abakumov E., Shtark O.Y., Zhukov V.A., Frolov A. (2023). Signaling in Legume–Rhizobia Symbiosis. Int. J. Mol. Sci..

[25] Venkateshwaran M., Volkening J.D., Sussman M.R., Ané J.M. (2013). Symbiosis and the Social Network of Higher Plants. Curr. Opin. Plant Biol..

[26] Wang J., Uggerhøj Andersen S., Ratet P. (2018). Molecular and Cellular Mechanisms of the Legume-Rhizobia Symbiosis. Front. Plant Sci..

[27] Favery B., Quentin M., Abad P. (2012). Compatible Plant-Root Knot Nematode Interaction and Parallels with Symbiosis. Signaling and Communication in Plant Symbiosis.

[28] Ghorbani S., Fernandez A., Hilson P., Beeckman T. (2014). Signaling Peptides in Plants. Cell Dev. Biol..

[29] Hu Z., Zhang H., Shi K. (2018). Plant Peptides in Plant Defense Responses. Plant Signal Behav..

[30] Xie H., Zhao W., Li W., Zhang Y., Hajný J., Han H. (2022). Small Signaling Peptides Mediate Plant Adaptions to Abiotic Environmental Stress. Planta.

[31] Schaller A. (2001). Bioactive Peptides as Signal Molecules in Plant Defense, Growth, and Development. Studies in Natural Products Chemistry.

[32] Takahashi F., Hanada K., Kondo T., Shinozaki K. (2019). Hormone-like Peptides and Small Coding Genes in Plant Stress Signaling and Development. Curr. Opin. Plant Biol..

[33] Wan X., Hontelez J., Lillo A., Guarnerio C., Van De Peut D., Fedorova E., Bisseling T., Franssen H. (2007). Medicago Truncatula ENOD40-1 and ENOD40-2 Are Both Involved in Nodule Initiation and Bacteroid Development. J. Exp. Bot..

[34] Luo Z., Wang J., Li F., Lu Y., Fang Z., Fu M., Mysore K.S., Wen J., Gong J., Murray J.D. (2023). The Small Peptide CEP1 and the NIN-like Protein NLP1 Regulate NRT2.1 to Mediate Root Nodule Formation across Nitrate Concentrations. Plant Cell.

[35] Van de Sande K., Pawlowski K., Czaja I., Wieneke U., Schell J., Schmidt J., Walden R., Matvienko M., Wellink J., Van Kammen A. (1996). Modification of Phytohormone Response by a Peptide Encoded by ENOD40 of Legumes and a Nonlegume. Science.

[36] Fang Y., Hirsch A.M. (1998). Studying Early Nodulin Gene ENOD40 Expression and Induction by Nodulation Factor and Cytokinin in Transgenic Alfalfa1. Plant Physiol..

[37] Kumagai H., Kinoshita E., Ridge R.W., Kouchi H. (2006). RNAi Knock-Down of ENOD40 s Leads to Significant Suppression of Nodule Formation in Lotus Japonicus. Plant Cell Physiol..

[38] Taleski M., Imin N., Djordjevic M.A. (2018). CEP Peptide Hormones: Key Players in Orchestrating Nitrogen-Demand Signalling, Root Nodulation, and Lateral Root Development. J. Exp. Bot..

[39] Chapman K., Ivanovici A., Taleski M., Sturrock C.J., Ng J.L.P., Mohd-Radzman N.A., Frugier F., Bennett M.J., Mathesius U., Djordjevic M.A. (2020). CEP Receptor Signalling Controls Root System Architecture in Arabidopsis and Medicago. New Phytol..

[40] Roy S., Griffiths M., Torres-Jerez I., Sanchez B., Antonelli E., Jain D., Krom N., Zhang S., York L.M., Scheible W.R. (2022). Application of Synthetic Peptide CEP1 Increases Nutrient Uptake Rates Along Plant Roots. Front. Plant Sci..

[41] Laffont C., Frugier F. (2024). *Rhizobium* Symbiotic Efficiency Meets CEP Signaling Peptides. New Phytol..

[42] Costa S.R., Ng J.L.P., Mathesius U. (2021). Interaction of Symbiotic Rhizobia and Parasitic Root-Knot Nematodes in Legume Roots: From Molecular Regulation to Field Application. Mol. Plant-Microbe Interact..

[43] Djordjevic M.A., Mohd-Radzman N.A., Imin N. (2015). Small-Peptide Signals That Control Root Nodule Number, Development, and Symbiosis. J. Exp. Bot..

[44] Valmas M.I., Sexauer M., Markmann K., Tsikou D. (2023). Plants Recruit Peptides and Micro RNAs to Regulate Nutrient Acquisition from Soil and Symbiosis. Plants.

[45] Pichon M., Journet E.P., Dedieu A., de Billy F., Truchet G., Barker D.G. (1992). *Rhizobium* Meliloti Elicits Transient Expression of the Early Nodulin Gene ENOD12 in the Differentiating Root Epidermis of Transgenic Alfalfa. Plant Cell.

[46] Crespi M.D., Jurkevitch E., Poiret M., d’Aubenton-Carafa Y., Petrovics G., Kondorosi E., Kondorosi A. (1994). Enod40, a Gene Expressed during Nodule Organogenesis, Codes for a Non-Translatable RNA Involved in Plant Growth. EMBO J..

[47] Sinvany G., Kapulnik Y., Wininger S., Badani H., Jurkevitch E. (2002). The Early Nodulin Enod40 Is Induced by; and Also Promotes Arbuscular Mycorrhizal Root Colonization. Physiol. Mol. Plant Pathol..

[48] Kouchi H., Hata S. (1993). Isolation and Characterization of Novel Nodulin CDNAs Representing Genes Expressed at Early Stages of Soybean Nodule Development. Mol. Gen. Genet..

[49] Yang W., Katinakis P., Hendriks P., Smolders A., de Vries F., Spee J., van Kammen A., Bisseling T., Franssen H. (1993). Characterization of GmENOD40, a Gene Showing Novel Patterns of Cell-specific Expression during Soybean Nodule Development. Plant J..

[50] Staehelin C., Charon C., Boller T., Crespi M., Kondorosi Á. (2001). Medicago Truncatula Plants Overexpressing the Early Nodulin Gene ENOD40 Exhibit Accelerated Mycorrhizal Colonization and Enhanced Formation of Arbuscules. Proc. Natl. Acad. Sci. USA.

[51] Röhrig H., Schmidt J., Miklashevichs E., Schell J., John M. (2002). Soybean ENOD40 Encodes Two Peptides That Bind to Sucrose Synthase. Proc. Natl. Acad. Sci. USA.

[52] Imin N., Mohd-Radzman N.A., Ogilvie H.A., Djordjevic M.A. (2013). The Peptide-Encoding CEP1 Gene Modulates Lateral Root and Nodule Numbers in Medicago Truncatula. J. Exp. Bot..

[53] Laffont C., Ivanovici A., Gautrat P., Brault M., Djordjevic M.A., Frugier F. (2020). The NIN Transcription Factor Coordinates CEP and CLE Signaling Peptides That Regulate Nodulation Antagonistically. Nat. Commun..

[54] Kereszt A., Mergaert P., Montiel J., Endre G., Kondorosi É. (2018). Impact of Plant Peptides on Symbiotic Nodule Development and Functioning. Front. Plant Sci..

[55] Batut J., Mergaert P., Masson-Boivin C. (2011). Peptide Signalling in the *Rhizobium*–Legume Symbiosis. Curr. Opin. Microbiol..

[56] Horváth B., Yeun L.H., Domonkos Á., Halász G., Gobbato E., Ayaydin F., Miró K., Hirsch S., Sun J., Tadege M. (2011). Medicago Truncatula IPD3 Is a Member of the Common Symbiotic Signaling Pathway Required for Rhizobial and Mycorrhizal Symbioses. Mol. Plant-Microbe Interact..

[57] Sun J., Miller J.B., Granqvist E., Wiley-Kalil A., Gobbato E., Maillet F., Cottaz S., Samain E., Venkateshwaran M., Fort S. (2015). Activation of Symbiosis Signaling by Arbuscular Mycorrhizal Fungi in Legumes and Rice. Plant Cell.

[58] Wang X., Feng H., Wang Y., Wang M., Xie X., Chang H., Wang L., Qu J., Sun K., He W. (2021). Mycorrhizal Symbiosis Modulates the Rhizosphere Microbiota to Promote Rhizobia–Legume Symbiosis. Mol. Plant.

[59] Mulder L., Hogg B., Bersoult A., Cullimore J.V. (2005). Integration of Signalling Pathways in the Establishment of the Legume-rhizobia Symbiosis. Physiol. Plant.

[60] Oldroyd G.E.D. (2013). Speak, Friend, and Enter: Signalling Systems That Promote Beneficial Symbiotic Associations in Plants. Nat. Rev. Microbiol..

[61] Stracke S., Kistner C., Yoshida S., Mulder L., Sato S., Kaneko T., Tabata S., Sandal N., Stougaard J., Szczyglowski K. (2002). A Plant Receptor-like Kinase Required for Both Bacterial and Fungal Symbiosis. Nature.

[62] Dávila-Delgado R., Flores-Canúl K., Juárez-Verdayes M.A., Sánchez-López R. (2023). Rhizobia Induce SYMRK Endocytosis in *Phaseolus vulgaris* Root Hair Cells. Planta.

[63] Das P.P., Singh K.R., Nagpure G., Mansoori A., Singh R.P., Ghazi I.A., Kumar A., Singh J. (2022). Plant-Soil-Microbes: A Tripartite Interaction for Nutrient Acquisition and Better Plant Growth for Sustainable Agricultural Practices. Environ. Res..

[64] Singh S., Parniske M. (2012). Activation of Calcium- and Calmodulin-Dependent Protein Kinase (CCaMK), the Central Regulator of Plant Root Endosymbiosis. Curr. Opin. Plant Biol..

[65] Pimprikar P., Carbonnel S., Paries M., Katzer K., Klingl V., Bohmer M.J., Karl L., Floss D.S., Harrison M.J., Parniske M. (2016). A CCaMK-CYCLOPS-DELLA Complex Activates Transcription of RAM1 to Regulate Arbuscule Branching. Curr. Biol..

[66] Cerri M.R., Wang Q., Stolz P., Folgmann J., Frances L., Katzer K., Li X., Heckmann A.B., Wang T.L., Downie J.A. (2017). The ERN1 Transcription Factor Gene Is a Target of the CCaMK/CYCLOPS Complex and Controls Rhizobial Infection in Lotus Japonicus. New Phytol..

[67] Soyano T., Hayashi M. (2014). Transcriptional Networks Leading to Symbiotic Nodule Organogenesis. Curr. Opin. Plant Biol..

[68] Rose C.M., Venkateshwaran M., Volkening J.D., Grimsrud P.A., Maeda J., Bailey D.J., Park K., Howes-Podoll M., den Os D., Yeun L.H. (2012). Rapid Phosphoproteomic and Transcriptomic Changes in the Rhizobia-Legume Symbiosis. Mol. Cell. Proteom..

[69] Singh S., Katzer K., Lambert J., Cerri M., Parniske M. (2014). CYCLOPS, A DNA-Binding Transcriptional Activator, Orchestrates Symbiotic Root Nodule Development. Cell Host Microbe.

[70] Pumplin N., Mondo S.J., Topp S., Starker C.G., Gantt J.S., Harrison M.J. (2010). Medicago Truncatula Vapyrin Is a Novel Protein Required for Arbuscular Mycorrhizal Symbiosis. Plant J..

[71] Bapaume L., Laukamm S., Darbon G., Monney C., Meyenhofer F., Feddermann N., Chen M., Reinhardt D. (2019). VAPYRIN Marks an Endosomal Trafficking Compartment Involved in Arbuscular Mycorrhizal Symbiosis. Front. Plant Sci..

[72] Feddermann N., Duvvuru Muni R.R., Zeier T., Stuurman J., Ercolin F., Schorderet M., Reinhardt D. (2010). The PAM1 Gene of Petunia, Required for Intracellular Accommodation and Morphogenesis of Arbuscular Mycorrhizal Fungi, Encodes a Homologue of VAPYRIN. Plant J..

[73] Guo D., Li J., Liu P., Wang Y., Cao N., Fang X., Wang T., Dong J. (2024). The Jasmonate Pathway Promotes Nodule Symbiosis and Suppresses Host Plant Defense in Medicago Truncatula. Mol. Plant.

[74] Samac D.A., Graham M.A. (2007). Recent Advances in Legume-Microbe Interactions: Recognition, Defense Response, and Symbiosis from a Genomic Perspective. Plant Physiol..

[75] Antolín-Llovera M., Petutsching E.K., Ried M.K., Lipka V., Nürnberger T., Robatzek S., Parniske M. (2014). Knowing Your Friends and Foes—Plant Receptor-like Kinases as Initiators of Symbiosis or Defence. New Phytol..

[76] Liu H., Zhang C., Yang J., Yu N., Wang E. (2018). Hormone Modulation of Legume-rhizobial Symbiosis. J. Integr. Plant Biol..

[77] Nakagawa T., Kawaguchi M. (2006). Shoot-Applied MeJA Suppresses Root Nodulation in Lotus Japonicus. Plant Cell Physiol..

[78] Ryu H., Cho H., Choi D., Hwang I. (2012). Plant Hormonal Regulation of Nitrogen-Fixing Nodule Organogenesis. Mol. Cells.

[79] Sun J., Cardoza V., Mitchell D.M., Bright L., Oldroyd G., Harris J.M. (2006). Crosstalk between Jasmonic Acid, Ethylene and Nod Factor Signaling Allows Integration of Diverse Inputs for Regulation of Nodulation. Plant J..

[80] Benjamin G., Pandharikar G., Frendo P. (2022). Salicylic Acid in Plant Symbioses: Beyond Plant Pathogen Interactions. Biology.

[81] Lian B., Zhou X., Miransari M., Smith D.L. (2000). Effects of Salicylic Acid on the Development and Root Nodulation of Soybean Seedlings. J. Agron. Crop Sci..

[82] Delay C., Imin N., Djordjevic M.A. (2013). CEP Genes Regulate Root and Shoot Development in Response to Environmental Cues and Are Specific to Seed Plants. J. Exp. Bot..

[83] Basile L.A., Lepek V.C. (2021). Legume–*Rhizobium* Dance: An Agricultural Tool That Could Be Improved?. Microb. Biotechnol..

[84] Hardarson G. (1993). Methods for Enhancing Symbiotic Nitrogen Fixation. Plant Soil.

[85] Dakora F.D., Phillips D.A. (2002). Root Exudates as Mediators of Mineral Acquisition in Low-Nutrient Environments. Plant Soil.

[86] Rodiño A.P., De La Fuente M., De Ron A.M., Lema M.J., Drevon J.J., Santalla M. (2011). Variation for Nodulation and Plant Yield of Common Bean Genotypes and Environmental Effects on the Genotype Expression. Plant Soil.

[87] Kipe-Nolt J.A., Giller K.E. (1993). A Field Evaluation Using the 15N Isotope Dilution Method of Lines of *Phaseolus vulgaris* L. Bred for Increased Nitrogen Fixation. Enhancement of Biological Nitrogen Fixation of Common Bean in Latin America.

[88] Nova-Franco B., Íñiguez L.P., Valdés-López O., Alvarado-Affantranger X., Leija A., Fuentes S.I., Ramírez M., Paul S., Reyes J.L., Girard L. (2015). The Micro-RNA172c-APETALA2-1 Node as a Key Regulator of the Common Bean-*Rhizobium etli* Nitrogen Fixation Symbiosis. Plant Physiol..

[89] Oladzad A., González A., Macchiavelli R., de Jensen C.E., Beaver J., Porch T., McClean P. (2020). Genetic Factors Associated with Nodulation and Nitrogen Derived from Atmosphere in a Middle American Common Bean Panel. Front. Plant Sci..

[90] Graham P.H., Rosas J.C. (1977). Growth and Development of Indeterminate Bush and Climbing Cultivars of *Phaseolus vulgaris* L. Inoculated with *Rhizobium*. J. Agric. Sci..

[91] Vargas M.A.T., Mendes I.C., Hungria M. (2000). Response of Field-Grown Bean (*Phaseolus vulgaris* L.) to *Rhizobium* Inoculation and Nitrogen Fertilization in Two Cerrados Soils. Biol. Fertil. Soils.

[92] Subramanian S., Stacey G., Yu O. (2007). Distinct, Crucial Roles of Flavonoids during Legume Nodulation. Trends Plant Sci..

[93] Mohd-Radzman N.A., Binos S., Truong T.T., Imin N., Mariani M., Djordjevic M.A. (2015). Novel MtCEP1 Peptides Produced in Vivo Differentially Regulate Root Development in Medicago Truncatula. J. Exp. Bot..

[94] Cantaro-Segura H., Huaringa-Joaquín A., Zúñiga-Dávila D. (2019). Symbiotic Effectiveness of Two *Rhizobium* Sp. Strains in Four Bean (*Phaseolus vulgaris* L.) Varieties in Peru. Idesia.

[95] Ogilvie H.A., Imin N., Djordjevic M.A. (2014). Diversification of the C-TERMINALLY ENCODED PEPTIDE (CEP) Gene Family in Angiosperms, and Evolution of Plant-Family Specific CEP Genes. BMC Genom..

[96] Ferguson B.J. (2013). Rhizobia and Legume Nodulation Genes. Brenner’s Encyclopedia of Genetics.

[97] Broughton W.J., Dilworth M.J. (1971). Control of Leghaemoglobin Synthesis in Snake Beans. Biochem. J..

[98] De Almeida Leite R., Martins L.C., Ferreira L.V.d.S.F., Barbosa E.S., Alves B.J.R., Zilli J.E., Araújo A.P., Jesus E.D.C. (2022). Co-Inoculation of *Rhizobium* and *Bradyrhizobium* Promotes Growth and Yield of Common Beans. Appl. Soil. Ecol..

