# Supplementary material for: Exogenous Application of ENOD40 and CEP1 Peptides Boosts Symbiotic Signaling Gene Expression and Productivity in Common Bean
Source: Plants (Basel). 2025 Sep 5;14(17):2786. doi: 10.3390/plants14172786 (PMC12431331; doi:10.3390/plants14172786)
Supplement: Supplementary file 1 [file plants-14-02786-s001.zip › plants-3628097-supplementary.pdf]

Supplementary Materials

Table S1. Primer information.

Gen	Genbank Number	Primer information used in the experimental process (F-R)		Source
ACTIN	XM_007139153.1	TGCAGAAGGTGAGGAGAGTTG	GGCAGAATGAACCAGTCAAAA	Reyero-Saavedra et al 2017
ENOD	X86441.1	GGGTCCTTACCCCTCACACT	TGTAGCCAAAGCCTCTCATCC	Reyero-Saavedra et al 2017
SYMRK	XM_007158267	GAAGATTTATGGTACTAGGT	TGTCAAGGCTACTCTGGA	Sanchez-Lopez et al., 2011
CCAMK	XM_007133469.1	GGCTCTTTCTCAAGGGAAGATAA	AGGTGGATACCCAGAGAGTAAG	Own elaboration
CYCLOPS	XM_007158089.1	ATCCAGCAGTGACCTGTTTC	CAGTATACCTCCGCCTCAATTC	Own elaboration
VAPYRIN	XM_007161918.1	CAGACCCGACCTAGTTCAATTAC	CAACGATCAGCTCTTCTCCTAAA	Own elaboration
AOS	XM_007160740	GGAAGGACCGTCAAGACTATTT	GAACGACGACGTTTGGTTTG	Own elaboration
ICS1	XM_007145542	CAAGGGACACCAAACTCTCTAC	CCTCTCTGCAACCGTTCATT	Own elaboration

Table S2. Three-way ANOVA for ENOD gene expression analysis.

Three-way ANOVA	Ordinary				
Alpha	0.05				
**Source of Variation**	**% of total variation**	**P value**	**P value summary**	**Significant?**	
Concentration	35.16	<0.0001	****	Yes	
Peptide	0.1435	0.4716	ns	No	
Inoculation	5.172	0.0001	***	Yes	
Concentration x Peptide	5.839	0.0008	***	Yes	
Concentration x Inoculation	29.52	<0.0001	****	Yes	
Peptide x Inoculation	2.764	0.0031	**	Yes	
Concentration x Peptide x Inoculation	12.75	<0.0001	****	Yes	
**ANOVA table**	**SS**	**DF**	**MS**	**F (DFn, DFd)**	**P value**
Concentration	157.8	3	52.59	F (3, 32) = 43.34	P<0.0001
Peptide	0.644	1	0.644	F (1, 32) = 0.5308	P=0.4716
Inoculation	23.21	1	23.21	F (1, 32) = 19.13	P=0.0001
Concentration x Peptide	26.21	3	8.736	F (3, 32) = 7.200	P=0.0008
Concentration x Inoculation	132.5	3	44.16	F (3, 32) = 36.40	P<0.0001
Peptide x Inoculation	12.4	1	12.4	F (1, 32) = 10.22	P=0.0031
Concentration x Peptide x Inoculation	57.22	3	19.07	F (3, 32) = 15.72	P<0.0001
Residual	38.83	32	1.213		

Table S3. Three-way ANOVA for SYMRK gene expression analysis.

Three-way ANOVA	Ordinary				
Alpha	0.05				
Source of Variation	% of total variation	P value	P value summary	Significant?	
Concentration	13.07	<0.0001	****	Yes	
Peptide	4.262	<0.0001	****	Yes	
Inoculation	54.99	<0.0001	****	Yes	
Concentration x Peptide	2.617	0.0067	**	Yes	
Concentration x Inoculation	6.615	<0.0001	****	Yes	
Peptide x Inoculation	2.791	0.0004	***	Yes	
Concentration x Peptide x Inoculation	9.923	<0.0001	****	Yes	
ANOVA table	SS	DF	MS	F (DFn, DFd)	P value
Concentration	525.6	3	175.2	F (3, 32) = 24.31	P<0.0001
Peptide	171.4	1	171.4	F (1, 32) = 23.78	P<0.0001
Inoculation	2212	1	2212	F (1, 32) = 306.9	P<0.0001
Concentration x Peptide	105.2	3	35.08	F (3, 32) = 4.867	P=0.0067
Concentration x Inoculation	266.1	3	88.69	F (3, 32) = 12.31	P<0.0001
Peptide x Inoculation	112.3	1	112.3	F (1, 32) = 15.58	P=0.0004
Concentration x Peptide x Inoculation	399.2	3	133.1	F (3, 32) = 18.46	P<0.0001
Residual	230.6	32	7.208		

Table S4. Three-way ANOVA for CCAMK gene expression analysis.

Three-way ANOVA	Ordinary				
Alpha	0.05				
Source of Variation	% of total variation	P value	P value summary	Significant?	
Concentration	49.81	<0.0001	****	Yes	
Peptide	0.006229	0.8847	ns	No	
Inoculation	16.17	<0.0001	****	Yes	
Concentration x Peptide	1.338	0.2258	ns	No	
Concentration x Inoculation	16.71	<0.0001	****	Yes	
Peptide x Inoculation	0.2390	0.3721	ns	No	
Concentration x Peptide x Inoculation	6.399	0.0007	***	Yes	
ANOVA table	SS	DF	MS	F (DFn, DFd)	P value
Concentration	4066	3	1355	F (3, 32) = 56.94	P<0.0001
Peptide	0.5084	1	0.5084	F (1, 32) = 0.02136	P=0.8847
Inoculation	1320	1	1320	F (1, 32) = 55.45	P<0.0001
Concentration x Peptide	109.2	3	36.40	F (3, 32) = 1.529	P=0.2258
Concentration x Inoculation	1364	3	454.6	F (3, 32) = 19.10	P<0.0001
Peptide x Inoculation	19.51	1	19.51	F (1, 32) = 0.8196	P=0.3721
Concentration x Peptide x Inoculation	522.3	3	174.1	F (3, 32) = 7.315	P=0.0007
Residual	761.6	32	23.80		

Table S5. Three-way ANOVA for CYCLOPS gene expression analysis.

Three-way ANOVA	Ordinary				
Alpha	0.05				
Source of Variation	% of total variation	P value	P value summary	Significant?	
Concentration	16.90	<0.0001	****	Yes	
Peptide	16.40	<0.0001	****	Yes	
Inoculation	3.186	0.0011	**	Yes	
Concentration x Peptide	12.13	<0.0001	****	Yes	
Concentration x Inoculation	19.49	<0.0001	****	Yes	
Peptide x Inoculation	0.3147	0.2660	ns	No	
Concentration x Peptide x Inoculation	23.72	<0.0001	****	Yes	
ANOVA table	SS	DF	MS	F (DFn, DFd)	P value
Concentration	3.046	3	1.015	F (3, 32) = 22.94	P<0.0001
Peptide	2.955	1	2.955	F (1, 32) = 66.77	P<0.0001
Inoculation	0.5742	1	0.5742	F (1, 32) = 12.97	P=0.0011
Concentration x Peptide	2.186	3	0.7288	F (3, 32) = 16.47	P<0.0001
Concentration x Inoculation	3.512	3	1.171	F (3, 32) = 26.45	P<0.0001
Peptide x Inoculation	0.05672	1	0.05672	F (1, 32) = 1.282	P=0.2660
Concentration x Peptide x Inoculation	4.276	3	1.425	F (3, 32) = 32.20	P<0.0001
Residual	1.416	32	0.04426		

Table S6. Three-way ANOVA for VAPYRIN gene expression analysis.

Three-way ANOVA	Ordinary				
Alpha	0.05				
Source of Variation	% of total variation	P value	P value summary	Significant?	
Concentration	30.78	<0.0001	****	Yes	
Peptide	0.1306	0.6463	ns	No	
Inoculation	19.55	<0.0001	****	Yes	
Concentration x Peptide	0.7027	0.7647	ns	No	
Concentration x Inoculation	4.631	0.0743	ns	No	
Peptide x Inoculation	8.212	0.0009	***	Yes	
Concentration x Peptide x Inoculation	16.50	0.0002	***	Yes	
ANOVA table	SS	DF	MS	F (DFn, DFd)	P value
Concentration	80.53	3	26.84	F (3, 32) = 16.85	P<0.0001
Peptide	0.3417	1	0.3417	F (1, 32) = 0.2146	P=0.6463
Inoculation	51.15	1	51.15	F (1, 32) = 32.12	P<0.0001
Concentration x Peptide	1.838	3	0.6127	F (3, 32) = 0.3847	P=0.7647
Concentration x Inoculation	12.11	3	4.037	F (3, 32) = 2.535	P=0.0743
Peptide x Inoculation	21.48	1	21.48	F (1, 32) = 13.49	P=0.0009
Concentration x Peptide x Inoculation	43.17	3	14.39	F (3, 32) = 9.036	P=0.0002
Residual	50.96	32	1.593		

Table S7. Three-way ANOVA for AOS gene expression analysis.

Three-way ANOVA	Ordinary				
Alpha	0.05				
Source of Variation	% of total variation	P value	P value summary	Significant?	
Concentration	2.106	0.1000	ns	No	
Peptide	8.418	<0.0001	****	Yes	
Inoculation	20.67	<0.0001	****	Yes	
Concentration x Peptide	38.87	<0.0001	****	Yes	
Concentration x Inoculation	3.226	0.0274	*	Yes	
Peptide x Inoculation	0.06185	0.6582	ns	No	
Concentration x Peptide x Inoculation	16.73	<0.0001	****	Yes	
ANOVA table	SS	DF	MS	F (DFn, DFd)	P value
Concentration	0.1079	3	0.03596	F (3, 32) = 2.264	P=0.1000
Peptide	0.4313	1	0.4313	F (1, 32) = 27.15	P<0.0001
Inoculation	1.059	1	1.059	F (1, 32) = 66.66	P<0.0001
Concentration x Peptide	1.991	3	0.6638	F (3, 32) = 41.78	P<0.0001
Concentration x Inoculation	0.1653	3	0.05510	F (3, 32) = 3.468	P=0.0274
Peptide x Inoculation	0.003169	1	0.003169	F (1, 32) = 0.1994	P=0.6582
Concentration x Peptide x Inoculation	0.8569	3	0.2856	F (3, 32) = 17.98	P<0.0001
Residual	0.5084	32	0.01589		

Table S8. Three-way ANOVA for ICS1 gene expression analysis.

Three-way ANOVA	Ordinary				
Alpha	0.05				
Source of Variation	% of total variation	P value	P value summary	Significant?	
Concentration	45.96	<0.0001	****	Yes	
Peptide	0.004455	0.9082	ns	No	
Inoculation	0.3366	0.3201	ns	No	
Concentration x Peptide	6.574	0.0013	**	Yes	
Concentration x Inoculation	25.15	<0.0001	****	Yes	
Peptide x Inoculation	0.3438	0.3151	ns	No	
Concentration x Peptide x Inoculation	11.07	<0.0001	****	Yes	
ANOVA table	SS	DF	MS	F (DFn, DFd)	P value
Concentration	90.81	3	30.27	F (3, 32) = 46.42	P<0.0001
Peptide	0.008802	1	0.008802	F (1, 32) = 0.01350	P=0.9082
Inoculation	0.6651	1	0.6651	F (1, 32) = 1.020	P=0.3201
Concentration x Peptide	12.99	3	4.330	F (3, 32) = 6.639	P=0.0013
Concentration x Inoculation	49.69	3	16.56	F (3, 32) = 25.40	P<0.0001
Peptide x Inoculation	0.6793	1	0.6793	F (1, 32) = 1.042	P=0.3151
Concentration x Peptide x Inoculation	21.86	3	7.287	F (3, 32) = 11.17	P<0.0001
Residual	20.87	32	0.6521		

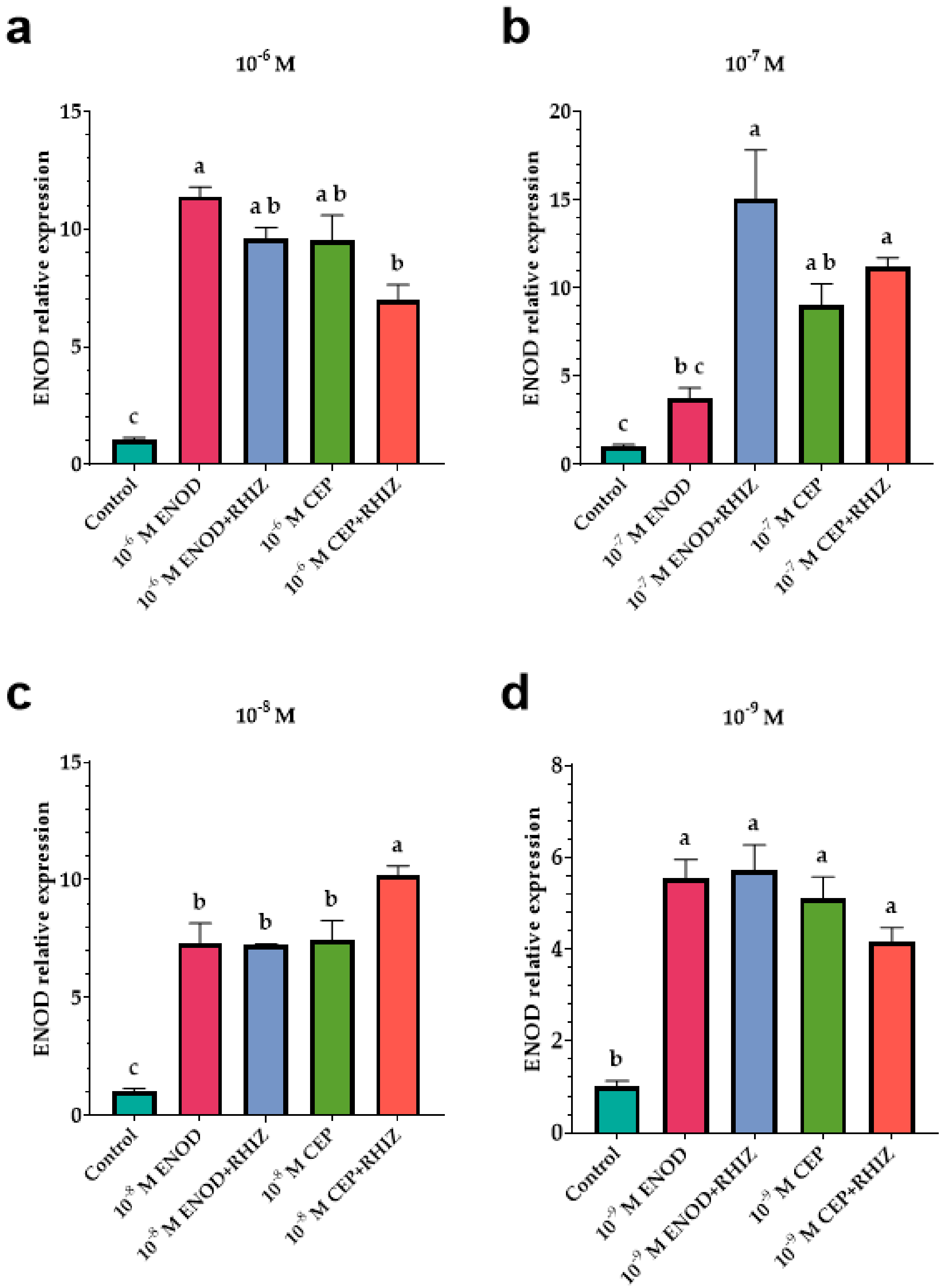

Figure S1. Relative expression of ENOD gene in *Phaseolus vulgaris* roots in response to different concentrations of ENOD40 and CEP1 peptides with and without *Rhizobium* inoculation: (a) 10^{-6} M , (b) 10^{-7} M, (c) 10^{-8} M, and (d) 10^{-9} M. Bars represent mean ± standard error of three biological replicates. Different letters indicating significant differences ($p < 0.05$, Tukey's HSD test).

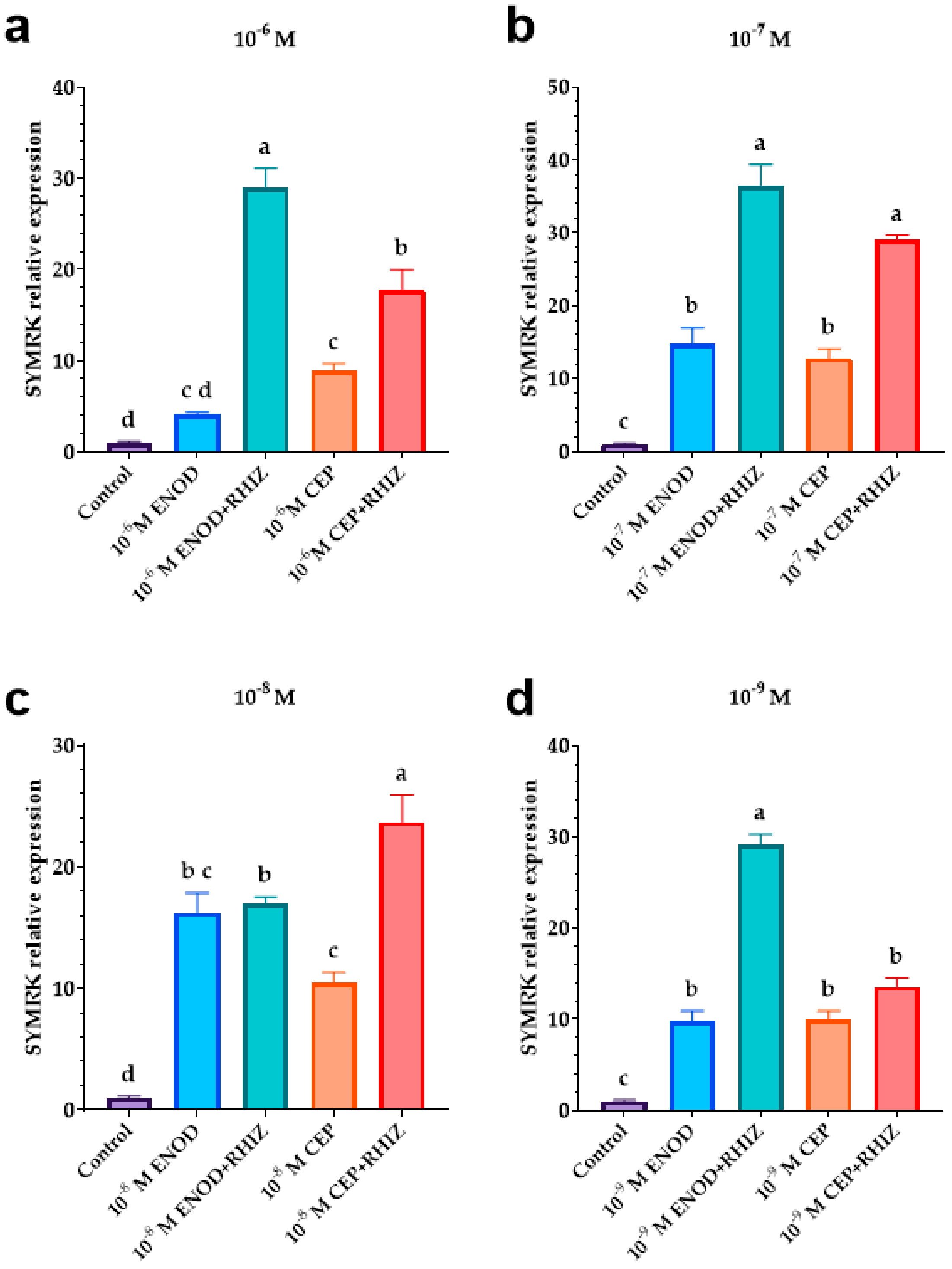

Figure S2. Relative expression of SYMRK gene in *Phaseolus vulgaris* roots in response to different concentrations of ENOD40 and CEP1 peptides with and without *Rhizobium* inoculation: (a) 10^{-6} M , (b) 10^{-7} M, (c) 10^{-8} M, and (d) 10^{-9} M. Bars represent mean ± standard error of three biological replicates. Different letters indicating significant differences ($p < 0.05$, Tukey's HSD test).

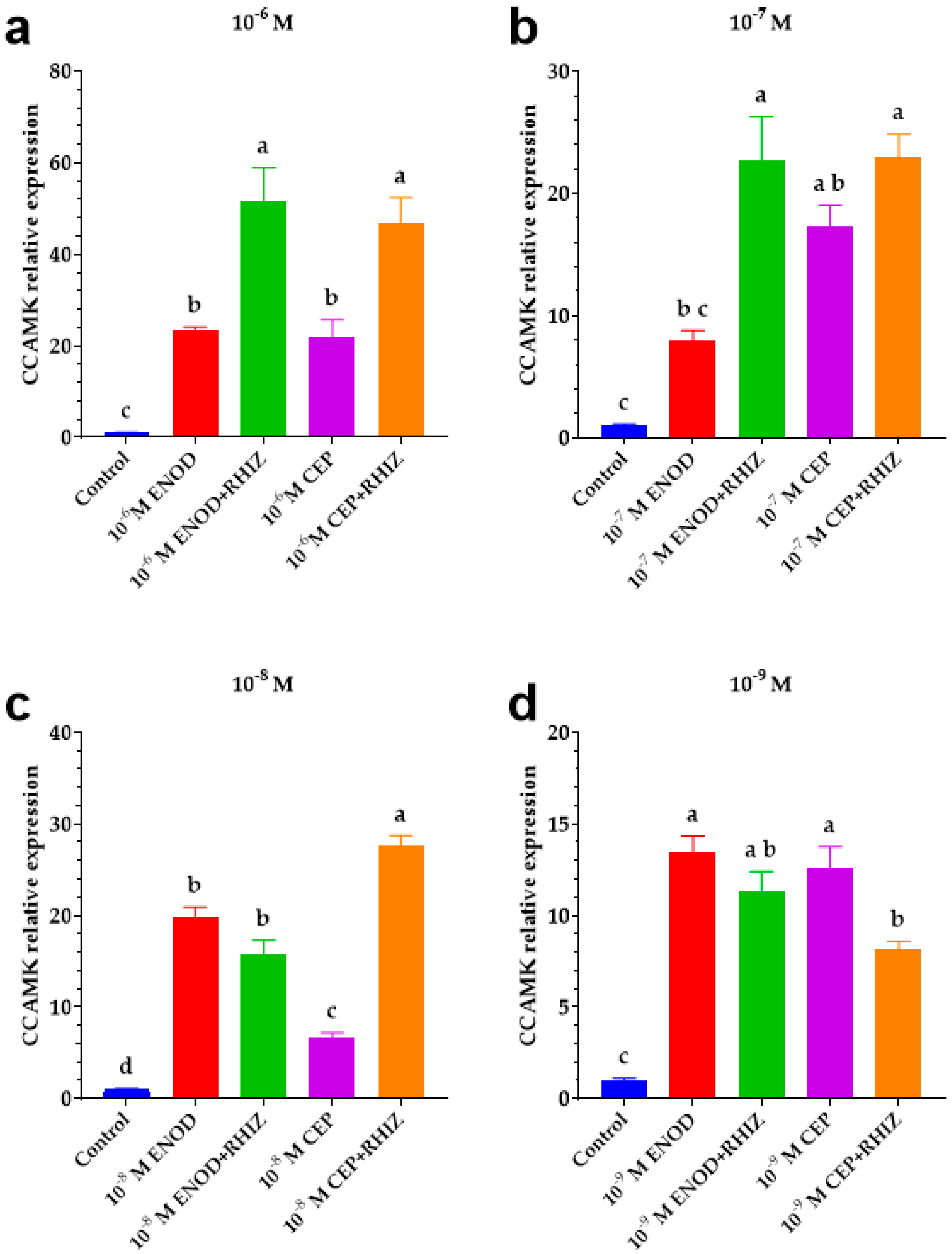

Figure S3. Relative expression of CCAMK gene in *Phaseolus vulgaris* roots in response to different concentrations of ENOD40 and CEP1 peptides with and without *Rhizobium* inoculation: (a) 10^{-6} M , (b) 10^{-7} M, (c) 10^{-8} M, and (d) 10^{-9} M. Bars represent mean ± standard error of three biological replicates. Different letters indicating significant differences ($p < 0.05$, Tukey's HSD test).

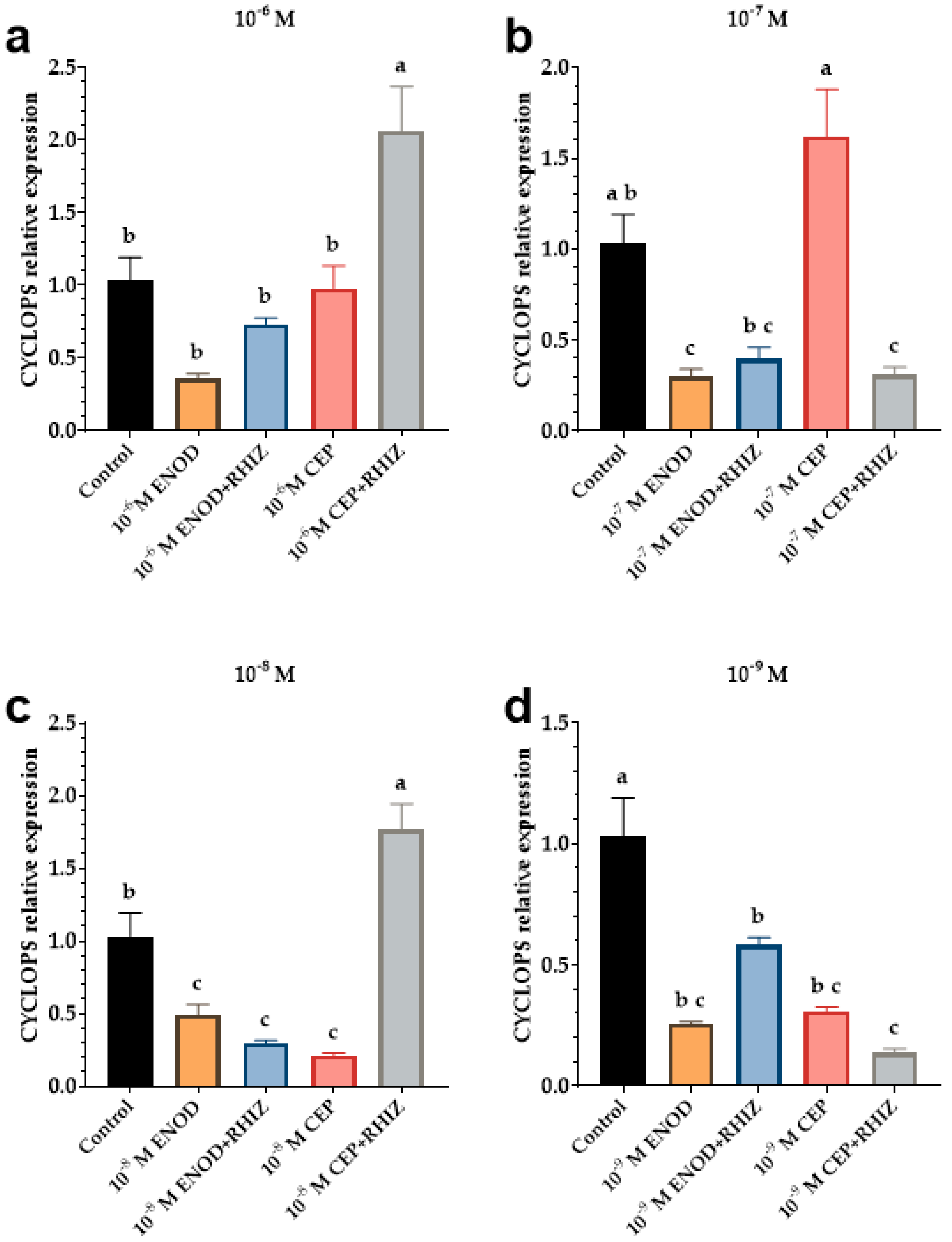

Figure S4. Relative expression of CYCLOPS gene in *Phaseolus vulgaris* roots in response to different concentrations of ENOD40 and CEP1 peptides with and without *Rhizobium* inoculation: (a) 10^{-6} M , (b) 10^{-7} M, (c) 10^{-8} M, and (d) 10^{-9} M. Bars represent mean ± standard error of three biological replicates. Different letters indicating significant differences ($p < 0.05$, Tukey's HSD test).

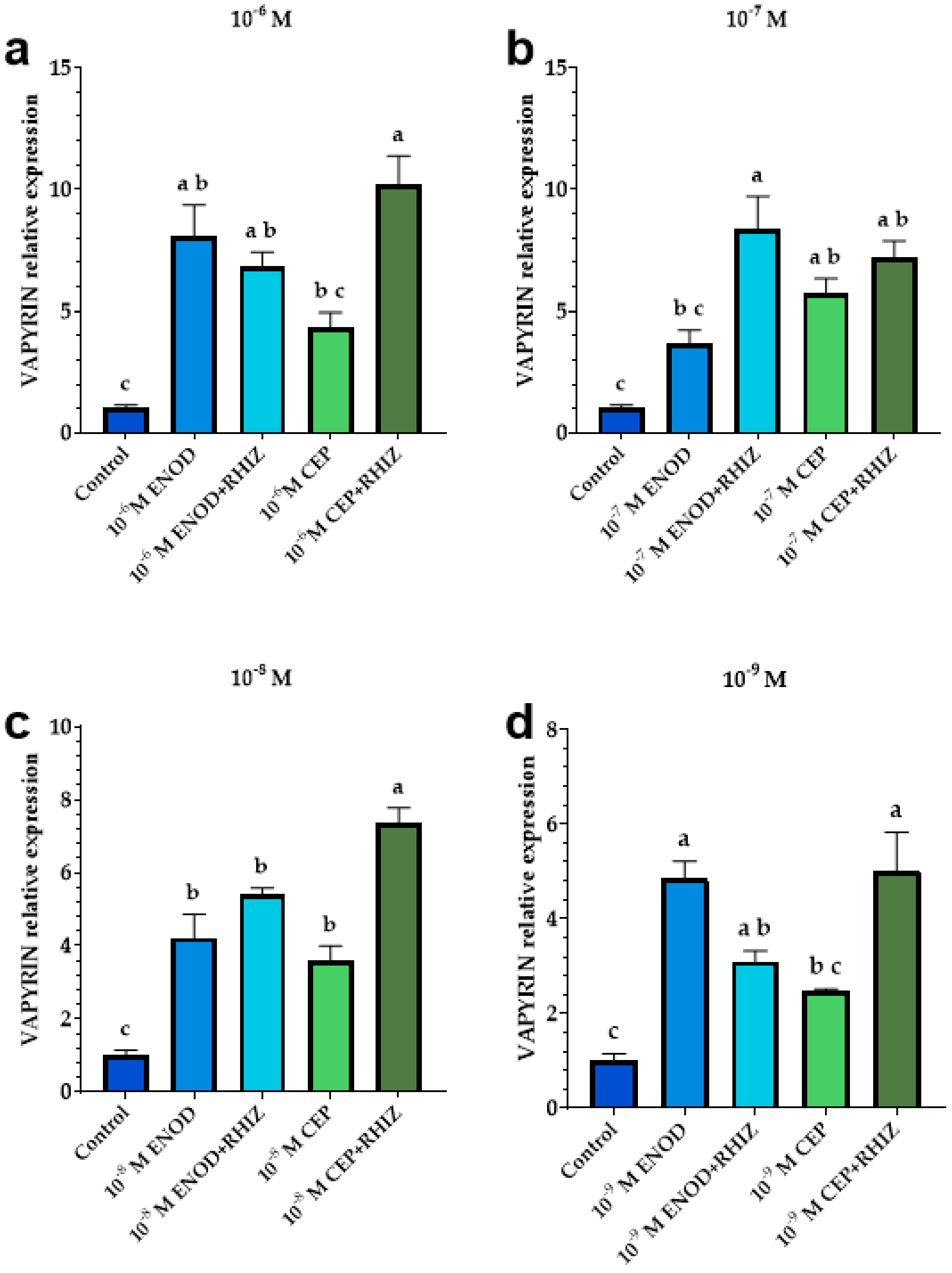

Figure S5. Relative expression of VAPYRIN gene in *Phaseolus vulgaris* roots in response to different concentrations of ENOD40 and CEP1 peptides with and without *Rhizobium* inoculation: (a) 10^{-6} M , (b) 10^{-7} M, (c) 10^{-8} M, and (d) 10^{-9} M. Bars represent mean ± standard error of three biological replicates. Different letters indicating significant differences ($p < 0.05$, Tukey's HSD test).

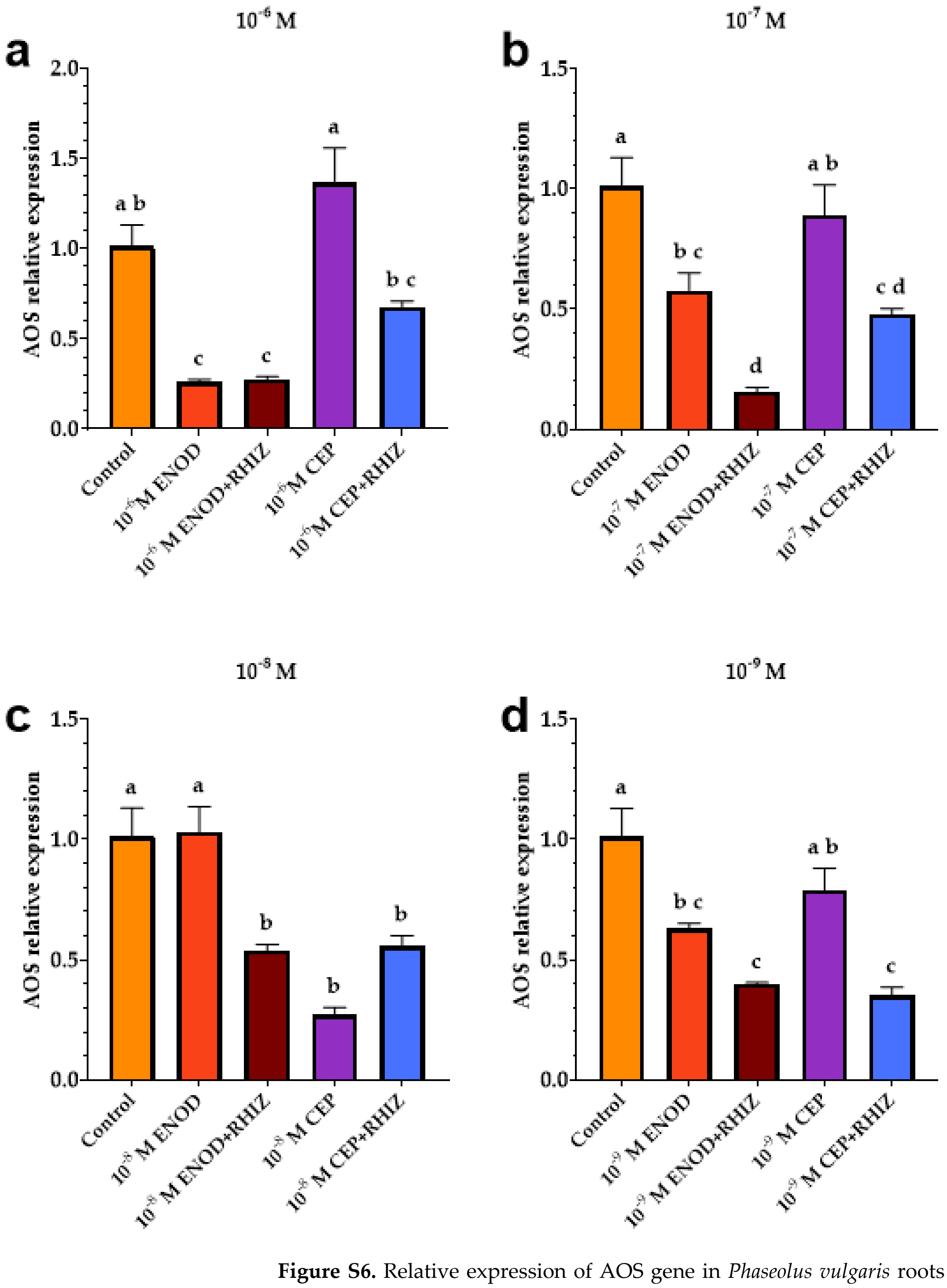

Figure S6. Relative expression of AOS gene in *Phaseolus vulgaris* roots in response to different concentrations of ENOD40 and CEP1 peptides with and without *Rhizobium* inoculation: (a) 10^{-6} M , (b) 10^{-7} M, (c) 10^{-8} M, and (d) 10^{-9} M. Bars represent mean ± standard error of three biological replicates. Different letters indicating significant differences ($p < 0.05$, Tukey's HSD test).

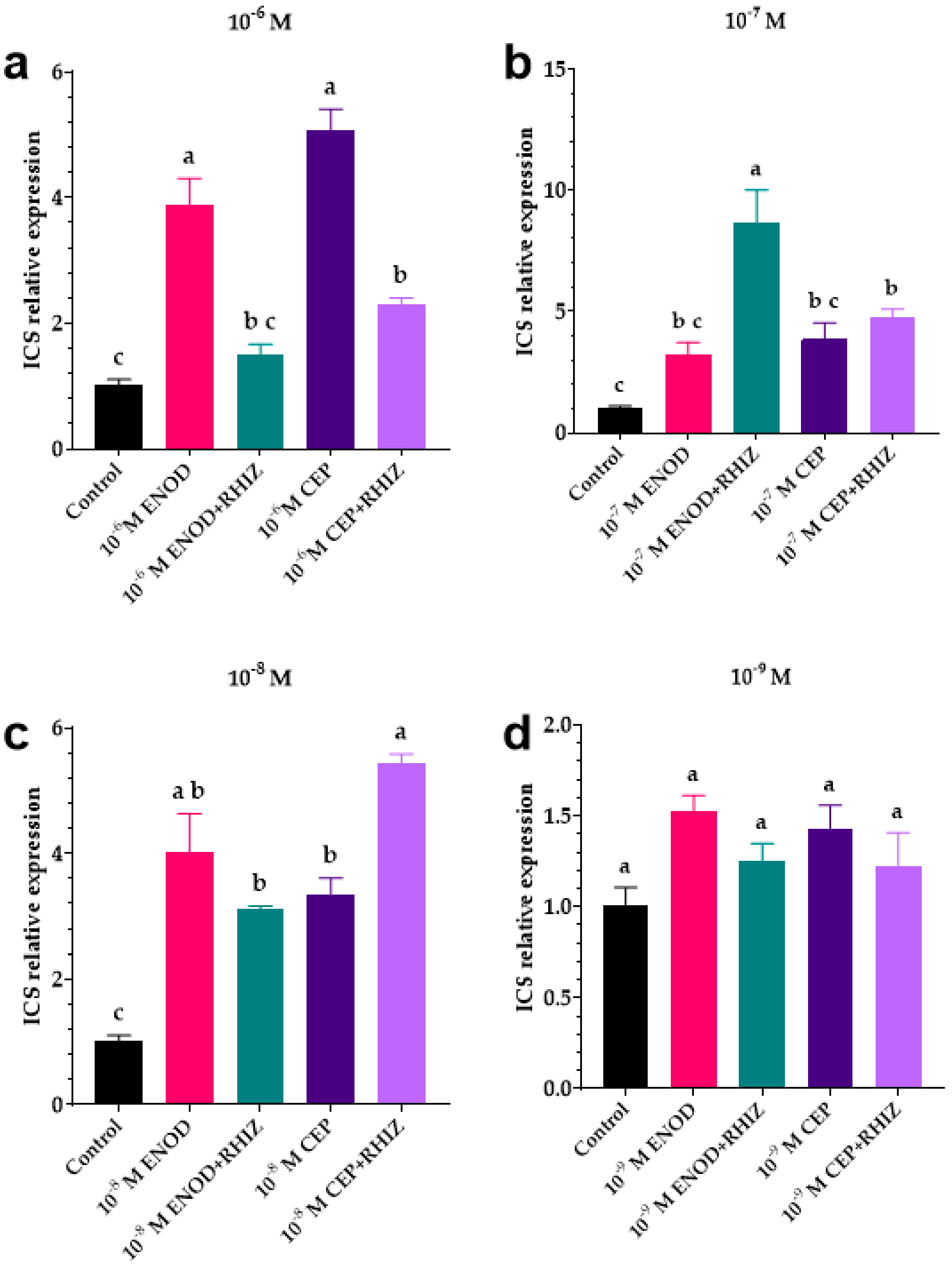

Figure S7. Relative expression of ICS1 gene in *Phaseolus vulgaris* roots in response to different concentrations of ENOD40 and CEP1 peptides with and without *Rhizobium* inoculation: (a) 10^{-6} M , (b) 10^{-7} M, (c) 10^{-8} M, and (d) 10^{-9} M. Bars represent mean ± standard error of three biological replicates. Different letters indicating significant differences ($p < 0.05$, Tukey's HSD test).

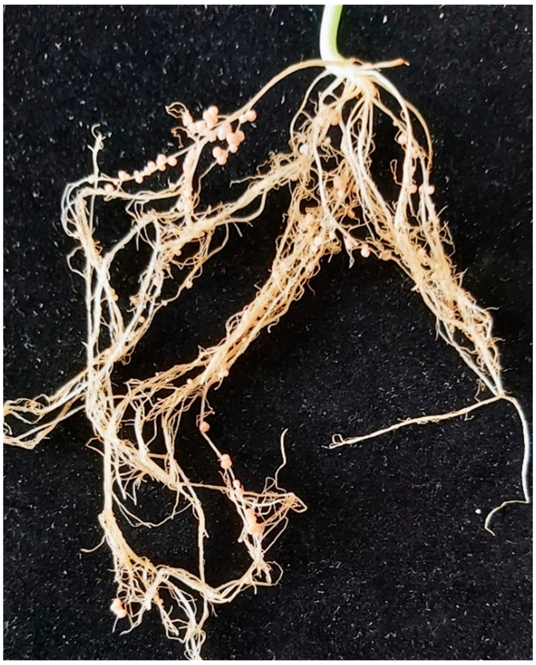

Figure S8. Common bean plant root sample applied with ENOD 10^{-7} M and inoculated with *Rhizobium*.

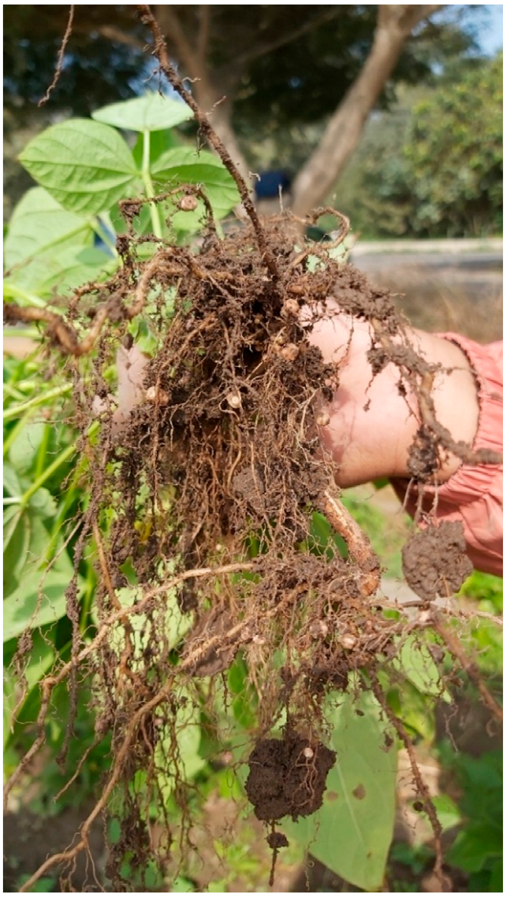

Figure S9. Common bean plant root harvested from the field with *Rhizobium* nodules.
